# Immuno-regulatory malignant B cells contribute to Chronic Lymphocytic Leukemia progression

**DOI:** 10.1038/s41417-023-00602-5

**Published:** 2023-03-28

**Authors:** Arsène Mékinian, Anne Quinquenel, Koceïla Ait Belkacem, Feriel Kanoun, Elisabetta Dondi, Emilie Franck, Marouane Boubaya, Maïssa Mhibik, Fanny Baran-Marszak, Rémi Letestu, Florence Ajchenbaum-Cymbalista, Vincent Lévy, Nadine Varin-Blank, Christine Le Roy

**Affiliations:** 1 INSERM, U978 Bobigny, France; 2 Université Paris 13 dite « Sorbonne Paris Nord », UFR SMBH, Labex INFLAMEX, Bobigny, France; 3 URC, APHP, Hôpital Avicenne, Bobigny, France; 4 Service d’Hématologie Biologique, APHP, Hôpital Avicenne, Bobigny, France; 5 CRC, APHP, Hôpital Avicenne, Bobigny, France

**Keywords:** Leukaemia, Cell biology, Tumour heterogeneity

## Abstract

Chronic Lymphocytic Leukemia (CLL) is a heterogeneous B cell neoplasm ranging from indolent to rapidly progressive disease. Leukemic cell subsets with regulatory properties evade immune clearance; however, the contribution of such subsets during CLL progression is not completely elucidated. Here, we report that CLL B cells crosstalk with their immune counterparts, notably by promoting the regulatory T (Treg) cell compartment and shaping several helper T (Th) subsets. Among various constitutively- and BCR/CD40-mediated factors secreted, tumour subsets co-express two important immunoregulatory cytokines, IL10 and TGFβ1, both associated with a memory B cell phenotype. Neutralizing secreted IL10 or inhibiting the TGFβ signalling pathway demonstrated that these cytokines are mainly involved in Th- and Treg differentiation/maintenance. In line with the regulatory subsets, we also demonstrated that a CLL B cell population expresses FOXP3, a marker of regulatory T cells. Analysis of IL10, TGFβ1 and FOXP3 positive subpopulations frequencies in CLL samples discriminated 2 clusters of untreated CLL patients that were significantly different in Tregs frequency and time-to-treatment. Since this distinction was pertinent to disease progression, the regulatory profiling provides a new rationale for patient stratification and sheds light on immune dysfunction in CLL.

## Introduction

Chronic Lymphocytic Leukemia (CLL), a neoplasm prevalent in the elderly, presents with a heterogeneous clinical course. While several patients exhibit indolent leukaemia that does not impact their life expectancy, others experience progressive disease with a rapid need for treatment [1]. In progressive CLL cases, clonal expansion of small mature B lymphocytes accumulates in the bone marrow and secondary organs. Despite the development of inhibitors and immunotherapies, treatments are not yet curative. The residual clonal cancer cells re-populate both lymphoid organs and peripheral blood, which is linked to a deficient antitumour immune surveillance [1, 2]. The heterogeneity of the CD5^+^CD19^+^ leukemic cells relies on the expression of phenotypic markers such as CD38, CD23, CD44, CD49d and CD27, stereotyped CDR3 sequences of immunoglobulin heavy chain variable region (IGHV) and genetic alterations at risk of progression [3–7]. Functionally, the heterogeneity also depends on antigen receptor (BCR)-triggered pathways leading to an apoptotic defect in progressive cases and an anergized phenotype for indolent cases [8–11].

CLL is also characterized by an imbalance of the subpopulations involved in immune surveillance and tumour recognition. T cell subsets, including CD4^+^ helpers (Th) and regulatory T cells (Tregs), have an altered ratio in this haematological malignancy [12, 13]. Thus, Tregs are increased in CLL and correlate with several clinical/biological features of progressive disease. Also, CD8^+^ T cells from CLL patients show functional defects in proliferation and cytotoxicity but preserve cytokine production reflecting T-cell exhaustion [14]. Ineffective anti-tumour immunity during neoplasm progression and production of several regulatory molecules and cytokines by specific B cell subsets ascribed to regulatory B cells lead to such impairment of immunological homeostasis [15–18]. These B cell subsets play an important role in the direct or indirect suppression of inflammatory response and the maintenance of tolerance. In peripheral blood from both healthy individuals and patients with autoimmune diseases or neoplasms, various IL10 producing subtypes have been reported [19–25]. Characterization of these subsets in murine models and human pathologies identified several phenotypes with suppressive activity without defining a unique consensus [17]. Among others, murine CD5^+^ B1a and CD1d^high^CD5^+^CD19^high^ B10 cells were IL10 producing cells [26, 27]. Studies unravelling the functional properties of the CD5^+^ B1a lineage have uncovered regulatory properties leading to a bias of the immune cell repertoire, including the expansion of the Treg population and suppression of Th1 and Th17 differentiation [28]. Subsets of human Breg cells can suppress Th1 differentiation and convert CD4^+^ T cells into Tregs *via* IL10 production [19, 29]. Induction of IL10 in various Breg subsets may require signals from activated CD4^+^ T cells with CD40L playing a significant stimulatory role [30]. In addition, induced Breg populations can also exert a suppressive mechanism *via* the production of TGFβ1, IL35 and indoleamine-2,3 dioxygenase (IDO) [31, 32], accounting also for Treg/Th17 balance and altered metabolism, respectively [33, 34]

Remarkably, various Breg populations express phenotypic markers commonly observed in CLL B-cell subsets, irrespective of their IGHV mutational status. Due to the expression of CD5, CLL cells have been hypothesized for a long time as being derived from a human B1 lineage recognizing natural antibodies [35, 36]. At present, CLL-B cells are considered antigen-experienced B cells with an IGHV mutational status, being either a T-dependent (mutated IGHV, M-IGHV) or T-independent (unmutated IGHV, UM-IGHV) memory phenotype with both subsets expressing CD27 [1]. CLL malignant B cells have a clear survival advantage over the other normal B cells [37]. Furthermore, diverse triggering events have been shown to induce IL10 in these cells, which share immunosuppressive capacities with B10 cells [24, 25].

In the present study, we first investigated the functional impact of CLL B cells on their immune counterparts ex-vivo and characterized the malignant secretome involved in tumour surveillance. Focusing on IL10 and TGFβ1-producing CLL subsets, which display a similar phenotypic signature, we found that the two cytokines can be co-expressed but are differentially regulated upon BCR/CD40L triggering. We also show that secreted IL10 and TGFβ1 have specific regulatory properties towards T cell differentiation and secretion. Importantly, we identified in a subset of leukaemic cells, the transcription factor FOXP3, a hallmark of Tregs and of several cancer cells. Finally, we demonstrate that an IL10-, TGFβ1- and FOXP3-expression signature allows the identification of two patient sub-groups with distinct time-to-treatment curves. These novel findings provide critical insight into how CLL cells modify their immunological environment during disease progression.

## Patients, materials and methods

### Patients

Blood samples were obtained during clinical follow-up of 97 CLL patients presenting at various stages of the disease after informed consent and approval by the local ethic committee (CLEA, GHPSSD, Avicenne hospital). CLL diagnosis was confirmed using international guidelines [38, 39] and investigators were blinded to the group allocation during the experiment and when assessing the outcome. Table 1 summarizes the clinical and biological parameters (Service d’Hématologie Biologique, Avicenne hospital), including age, sex, Binet stage and IGHV mutational status at the experimental time, as well as treatment status for the cohorts of patients; Supplementary Tables 1, 2 provide biological and clinical parameters for cohorts 1 and 2.

**Table 1:** Patients’ biological and clinical parameters.

Numbers of CLL samples	*n* = 97
Sex (Female/Male ratio)	34 / 63 (35.05% / 64.95%)
Age (years)	Mean: 66.91
	Median: 66
IGHV mutational status	
Unmutated:	39 (40.20%)
Mutated:	55 (56.70%)
NA:	3 (3.10%)
Binet Stage at the experiment time (number and %)	
A:	61/97 (62.89%)
B:	14/97 (14.43%)
C:	10/97 (10.30%)
ND:	12/97 (12.37%)
Treatment (more than 2 years prior to the experiment time)	
Cohort #1 (*n* = 28)	
Cluster 1 (*n* = 17; 4 NA)	2/13 (15.38%)
Cluster 2 (*n* = 11; 1 NA)	5/10 (50.00%)
Need of Treatment	
Cohort #2 (*n* = 23)	
Cluster 1 (*n* = 15)	5/15 (33.33%)
Cluster 2 (*n* = 8)	5/8 (62.50%)

*ND* Not Determined, *NA* Not Available.

### Human cells isolation, cells lines and cell cultures

Peripheral Blood Mononuclear Cells (PBMCs) were isolated by density gradient. Lymphocytes were isolated from total blood by negative selection using Rosette B and CD4^+^ isolation kits (STEMCELL Technologies, Grenoble, France). PBMCs, isolated B and T cells subsets purity was assessed by flow cytometry and were typically >95% pure. Cell viability was quantified with the Vi-CELL^TM^ XR Cell Viability Analyzer based on the Trypan Blue Exclusion method (Beckman Coulter, Life Sciences, Villepinte, France). All cells (4 × 10^6^ cells/ml) were cultured in RPMI 1640 containing L-glutamine, supplemented with 100 U/mg/ml penicillin/streptomycin and 10% FCS (PAA, Les Mureaux, France) at 37 °C and 5% CO2 for up to 72 h. PBMCs and B cells were treated for 48 h with blocking antibodies (Abs): rat anti-IL10 (JES3-9D7, Becton Dickinson, Pont-de-Claix, France), rat anti-IL10 receptor (3F9, BioLegend, Paris, France) or with the selective inhibitor of TGFβRI (SB 431542, Bio-Techne, TOCRIS, Noyal Châtillon, France). B cells were stimulated or not with a combination of soluble CD40L (1 µg/ml; Miltenyi Biotec, Paris, France) and coated anti-IgM (20 µg/ml; Jackson ImmunoResearch, Montlucon, France), a combination of soluble CD40L and IL21 (50 ng/ml, ThermoFisher Scientific, Les Ulis, France), human recombinant IL10 (40 ng/ml) or TGFβ1 (5 ng/ml) (Miltenyi Biotec). CD4^+^ T cells were stimulated or not on coated anti-CD3 mAb (10 µg/ml; Hit-3a, Ebioscience, ThermoFisher Scientific) and anti-CD28 mAb (1 µg/ml; CD28-2, Ebiosciences) plates. For cytokine detection by flow cytometry, PIB treatment - PMA (500 ng/ml), Ionomycin (1 µg/ml) and Brefeldin A (BFA) (10 µg/ml) (Sigma Aldrich, Saint Quentin Fallavier, France)- was added for the last 4 h of culture. Co-culture experiments were performed with purified autologous CD4^+^ T / B cells at various ratios (1:1, 1:2, 1:5 and 1:10) with a total of 2 × 10^6^ cells for 3 days in the presence or not of CD3/CD28 and/or α-IgM/CD40L stimulation. U2OS cells (HTB-96, ATCC) and HEK293T cells (CRL-3216, ATCC) were cultured and tested for mycoplasma contamination, respectively, in McCoy’s 5 A or DMEM supplemented with 10 % FCS and 100 U/mg/ml penicillin/streptomycin.

Detailed protocols used in the following sections are described in the Supplementary Information. Antibodies and probes are listed in the Supplementary Table 3.

### Cell sorting

After doublet exclusion, CD5^+^CD19^+^ cells purified from CLL peripheral blood were sorted further by gating for CD5^+^, CD19^+^, CD3^−^ and CD27^high^ cells on a FACS ARIA III cell sorter (BD Bioscience).

### Western blotting

Total protein lysates (2 to 50 μg) were separated on 10% SDS-PAGE, transferred on a nitrocellulose membrane and incubated with the indicated antibodies followed by incubation with appropriate secondary horseradish peroxidase-conjugated antibodies. Detection was performed using ECL kit (Bio-Rad, Marnes-la-Coquette, France) and images were acquired with a Chemidoc MP (Bio-Rad).

### Flow cytometry

Freshly isolated PBMCs, leukaemic B cells, CD4^+^ T in culture or in co-culture for 0, 2 or 3 days, were stimulated or not with anti-IgM/CD40L, or treated or not with anti-IL10 or TGFβRI inhibitor, and/or with PIB. Cells were stained for viability, extracellularly labelled with antibodies for CD antigens, fixed and permeabilized, intracellularly stained for cytokines and/or FOXP3 according to manufactures’ instructions. After washes, cells were analyzed on FACSCanto II or Symphony^TM^ A3 SORP analyzer (Becton Dickinson) driven by the BD FACSDIVA^TM^ software and data compiled with the FlowJo^TM^ software (BD Biosciences). To detect RNA targets, B cells or PBMCs were processed according to the manufacturer’s instructions (PrimeFlow RNA assay, Invitrogen, ThermoFisher Scientific) using target-specific probe sets.

### Quantification of cytokine secretion

Cytokines (IL-1β, IL2, IL4, IL6, IL8, IL10, IL13, IL17, TNFα, IFNγ and TGFβ1) were quantified in the supernatants of CLL B cells cultured and stimulated or not for 72 h by V-plex assays (MSD) according to the manufacturer’s protocols.

### Statistical analyses

Data are expressed as means ± SEM or frequencies. For the comparison, Mann—-Whitney U test or Wilcoxon signed-rank test (paired data) were used. The Spearman’s rank correlation coefficient test was used to assess the relationship between pairs of secreted cytokines. Unsupervised hierarchical cluster analysis was performed using IL10 and TGFβ1 and/or FOXP3 percentages of expressing cells. The distribution of Time-to-Treatment was estimated using the Kaplan-Meier method. The difference between clusters was tested by Log-rank. All tests were two-sided at a 0.05 significance level and the variance was similar between the groups that were compared. Analyses were performed using the R statistical software version 3.1.2 and Graphpad (Prism 7).

## Results

### CLL cells crosstalk with their immune counterparts

**Fig. 1:**
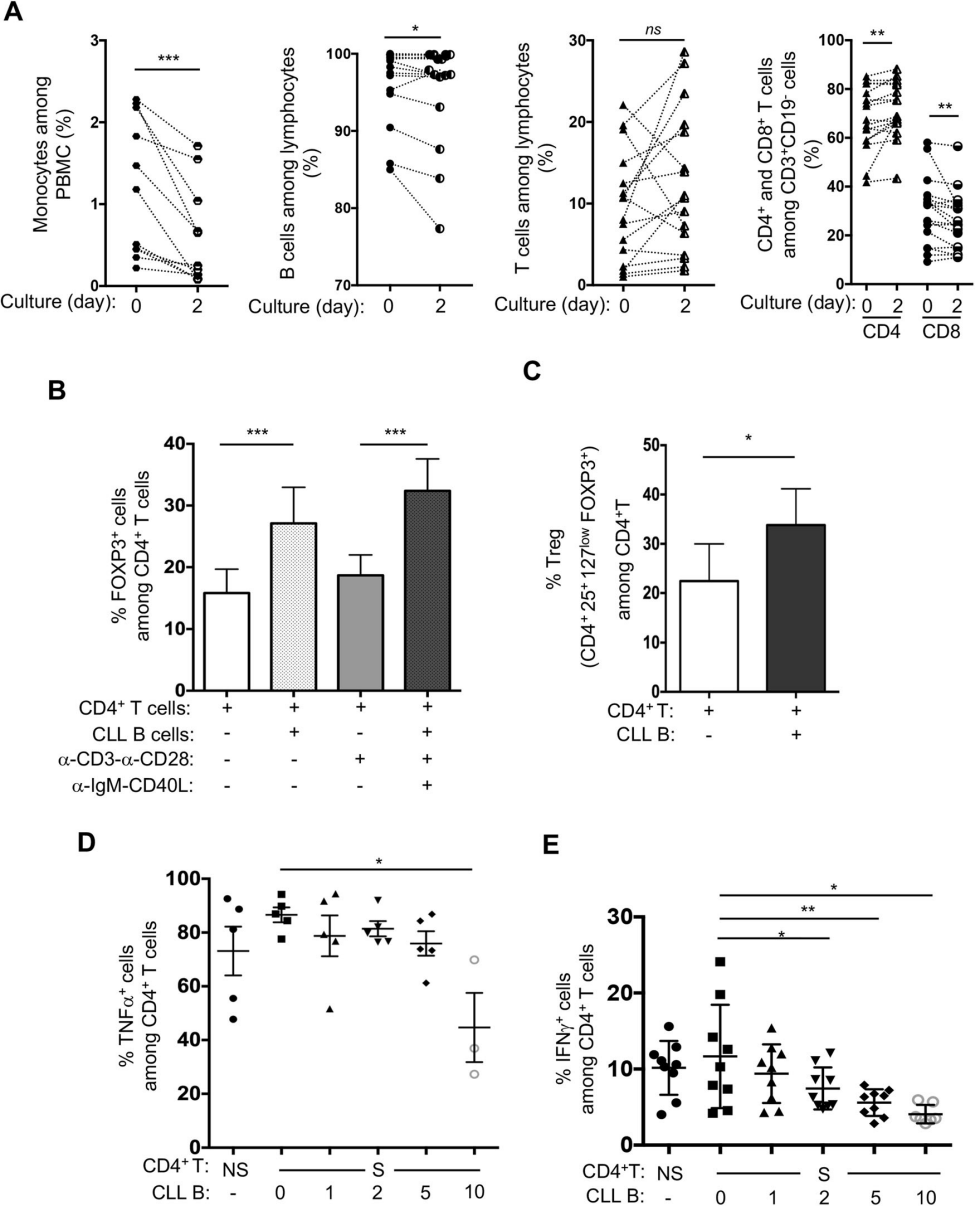
CLL B cells undertake a regulatory crosstalk with their immune counterparts. **A** Frequencies of various cell subsets (monocytes, *n* = 11; leukemic B cells, *n* = 17; T cells, *n* = 16; CD4^+^/CD8^+^ T cells, *n* = 17) were evaluated by flow cytometry from CLL patients PBMCs prior to or upon 2 days of culture. Dotted lines link individual patient samples. Wilcoxon signed-rank test on means *P ≤ 0.05, **P ≤ 0.01, ***P ≤ 0.001, *ns* not significant. **B**–**E** Purified autologous CD4^+^ T cells stimulated with anti-CD3 and anti-CD28 (+, S) or not (−, NS) were cultured for 2 days in with (+) or without (−) CLL B cells activated (+) or not (−) with CD40L and anti-IgM as indicated and analyzed by flow cytometry. Frequencies of FOXP3^+^ cells (**B**), of CD4^+^CD25^+^CD127^low^FOXP3^+^ Treg cells (**C**) out of CD4^+^ T cells in the culture are graphed (T/B ratio 1:1; *n* = 5). Autologous CLL CD4^+^ T and B cells were cultured for 48 h at the indicated ratios (T/B). Scatter dot plots indicate the frequencies of TNFα^+^ (**D**; *n* = 5) and IFNγ^+^ (**E**; *n* = 9) cells out of CD4^+^ T cells. ANOVA test *P < 0.01; **P < 0.001.

To determine whether CLL cells may impact other immune cells, PBMCs from CLL patients were cultured for two days and subsequently evaluated by flow cytometry to determine the proportion of various immune subsets. Monocytes (CD14^+^CD16^+^/CD14^+^CD16^-^/CD14^-^CD16^+^) (mean 1.20 ± 0.25 at t = 0 *vs* 0.59 ± 0.18 at t = 2 days, *n* = 11), as well as CLL B cells, decreased in most of the samples tested (99.1 ± 1.19 *vs* 97.9 ± 1.60, *n* = 17). While the overall percentage of T cells remained in the same range during the 2 days-culture (9.57 ± 1.70 *vs* 12.59 ± 2.20, *n* = 16), the proportion of CD4^+^ or CD8^+^ T cells increased or decreased, respectively (CD4: 67.98 ± 3.18 *vs* 71.71 ± 2.76; CD8: 29.83 ± 3.36 *vs* 26.01 ± 2.89, *n* = 17) (Fig. 1A and S1A). To further characterize this modulation, autologous purified CD4^+^ T cells and CLL B cells were cultured separately or together, and the proportion of CD4^+^ T cells expressing FOXP3 was evaluated. The autologous co-culture increased the number of FOXP3^+^ regulatory T cells. In coculture, BCR-mediated stimulation of B cells led to an increase of the Tregs population while TCR-mediated stimulation of T cells alone did not (Fig. 1B). The CD4^+^CD25^+^CD127^low^FOXP3^+^ Tregs enhancement confirmed the increase of FOXP3^+^ T cells in the presence of B cells (Fig. 1C). Moreover, the co-culture of autologous CD4^+^ and B cells strongly impacted T cell capacity to produce two important pro-inflammatory type 1 cytokines: TNFα and IFNγ. This regulatory effect was dependent on the ration of T/B cells (1/1 to 1/10) present in the co-culture (Fig. 1D, E, S1B, C). Altogether these results assessed the regulatory function of CLL B cells with a suppressive activity on Th1 cellular response and the induction of CD4^+^ Tregs.

### Specific subsets of CLL B cells express IL10 together with TGFβ1

**Fig. 2:**
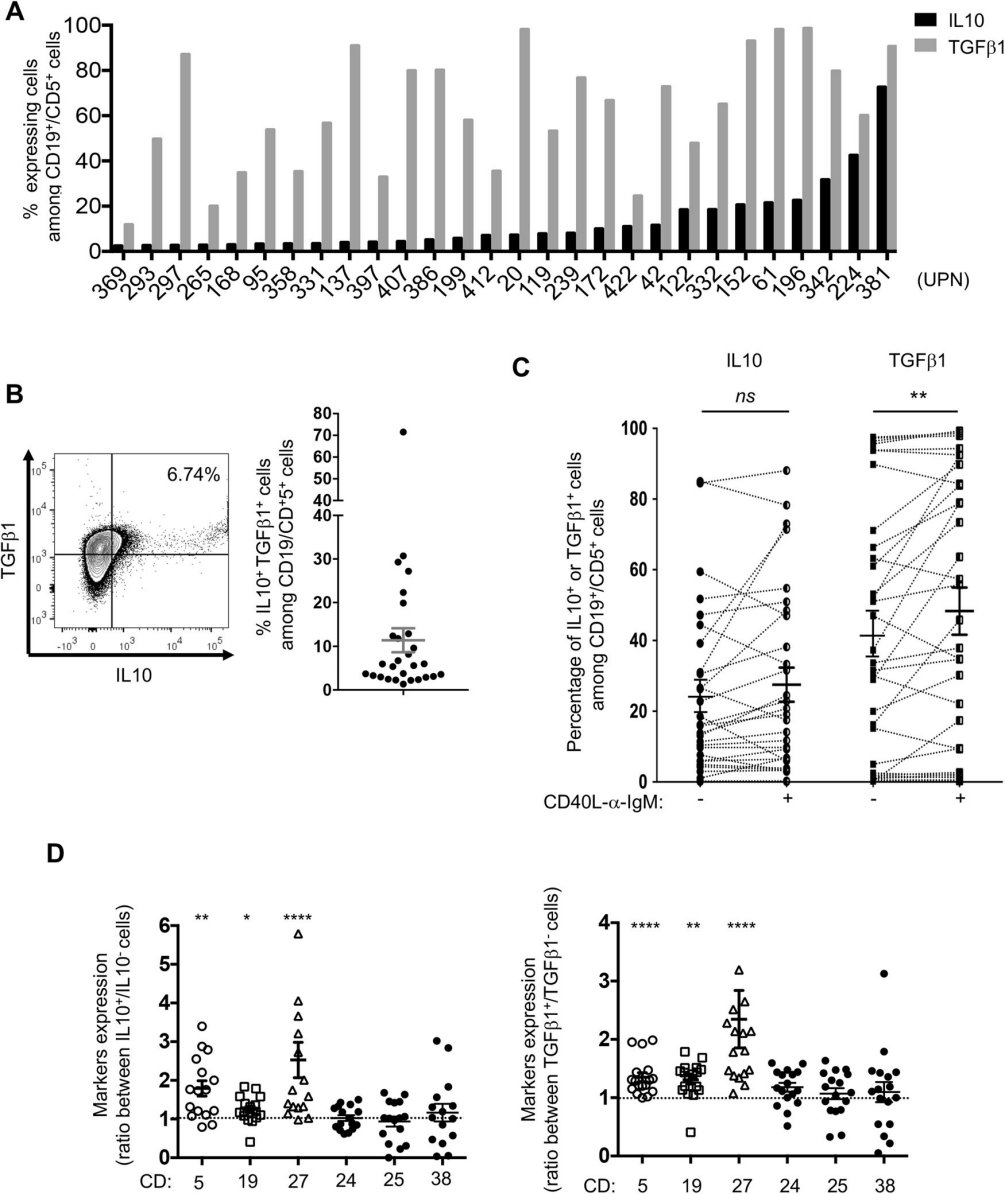
Specific subsets of CLL B cells express IL10 together with TGFβ1. **A** Percentage of IL10-(black bars) or TGFβ1-(grey bars) expressing cells among CD19^+^ CD5^+^ cells from patient samples (UPN) were evaluated by flow cytometry and graphed; (*n* = 28). **B** Representative dot plot showing IL10^+^ and TGFβ1^+^ staining in CD19^+^/CD5^+^ cells from a patient PBMC sample (bars indicate the respective control isotypes, UPN 239, left). Graphic representation of the frequency of CLL B cells co-expressing IL10 and TGFβ1 among PBMCs from patients’ samples (right). **C** Graphic representation of IL10^+^ or TGFβ1^+^ purified CLL B cells frequencies. Cells were stimulated or not with CD40L/ anti-IgM for 3 days (IL10 *n* = 29, TGFβ1 *n* = 33) and quantified by flow cytometry. Dotted lines link individual patient samples. Paired *t*-test, **P ≤ 0.01, *ns*, not significant *P* > 0.05. **D** Purified B cells were cultured for 3 days, labelled with the indicated membrane markers and stained for cytoplasmic IL10 or TGFβ1. MFI ratio of CD5, 19, 27, 24, 25, 27 and 38 between IL10 positive versus negative (*n* = 16, left) or TGFβ1 positive versus negative (*n* = 17, right) cells are graphed. Wilcoxon signed-rank test *P ≤ 0.05, **P ≤ 0.01, ****P ≤ 0.0001.

Based on the regulatory function of CLL B cells observed, we next evaluated the expression of two major immunosuppressive cytokines, IL10 and TGFβ1, in a cohort of 28 patients. Flow cytometry analysis of CD5^+^CD19^+^ cells (Fig. S2A) in PBMC samples showed a highly variable proportion of cells expressing IL10 or TGFβ1 (Fig. 2A), with several of them expressing both cytokines (Fig. 2B). The variable expression and co-expression of IL10 and TGFβ1 were confirmed at the transcriptional level using RNA-Flow cytometry analysis, (Fig. S2B). We also examined whether IL10 and TGFβ1 production in purified CLL B cells was regulated by anti-IgM/CD40L. Although several patient samples showed a more pronounced positive or negative modulation, comparable frequencies of IL10^+^ cells were found with or without anti-IgM/CD40L stimulation (*n* = 30, ranging upon stimulation from 0.12 to 88.1 %, mean 28.12 ± 4.85%). By contrast, the overall proportion of TGFβ1 expressing cells was significantly increased upon triggering (*n* = 33, ranging from 0.28 to 99.3 % *vs* 0.22 to 97.5%, mean 48.45 ± 6.67% *vs* 42.26 ± 6.39%) (Fig. 2C, connected lines, and Fig. S2C), suggesting that BCR-mediated activation may be involved in the modulatory function of CLL B cells. Of note several samples containing very low proportions of TGFβ1^+^ cells remained at similar low expression upon BCR triggering.

The phenotypic signature of IL10- or TGFβ1-positive subsets was specified by flow cytometry using different phenotypic markers previously described in various regulatory populations (CD5, CD19, CD27, CD24, CD25 and CD38). IL10^+^ and IL10^-^ cells exhibited significantly different expression levels of CD5, CD19 and CD27 (Fig. 2D). This difference in expression was also observed in TGFβ1^+^ and TGFβ1^-^ cells without any stimulation. Stimulation with anti-IgM/CD40L resulted in similar expression profiles (Fig. S2D). As already described for CLL clonality, phenotyping of IL10^+^ or TGFβ1^+^ subsets suggested that both belong to memory B cells. IL21/CD40L stimulation significantly increased the expression of Granzyme B (GrB) in B cells from healthy subjects, whereas it remained weakly expressed in CLL patients regardless of stimulation (Fig. S2E).

### CLL B cells secrete IL10, TGFβ1 and other soluble factors

**Fig. 3:**
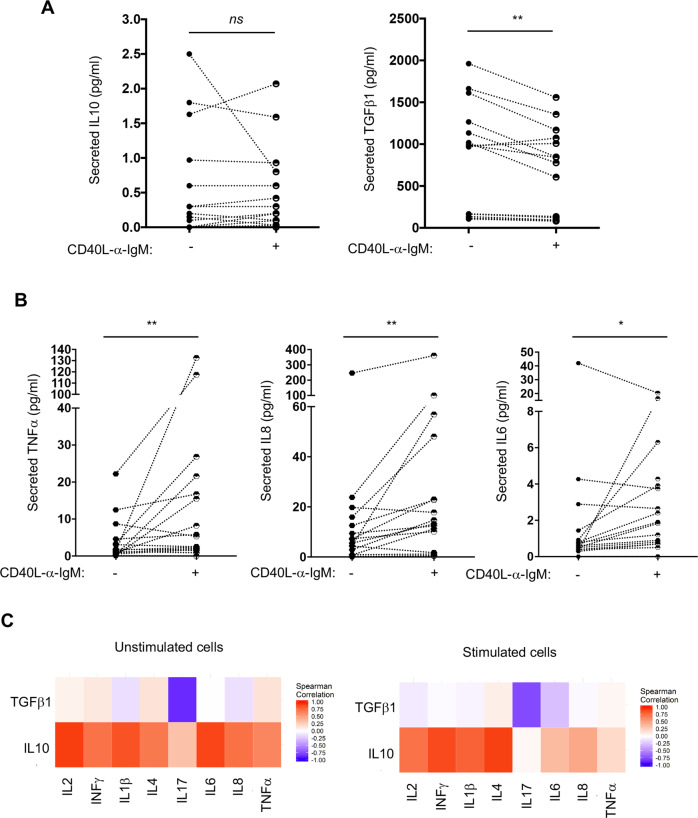
CLL B cells secrete IL10, TGFβ1 as well as others soluble factors. Graphic representation of IL10 or TGFβ1 secreted levels (**A**), TNFα, IL8 or IL6 (**B**) evaluated by “multi-ELISA”-like technology (*n* = 17) from the culture supernatant of purified CD5^+^CD19^+^ cells stimulated (+) or not (−) with CD40L/anti-IgM for 72 h. Wilcoxon matched-paired signed rank test, *P ≤ 0.05; **P ≤ 0.01. **C** A Spearman correlation was established between the levels of IL10 or TGFβ1 and various cytokines secreted in the supernatant of cells stimulated (right) or not (left) for 72 h.

The capacity of CLL B cells to secrete various cytokines was evaluated using a multiplex ELISA-like quantification of the leukemic cell supernatants following 3 days of culture with or without CD40L/anti-IgM stimulation. IL10 was detected at a much lower level than TGFβ1. While CD40L/anti-IgM stimulation did not modulate IL10 levels, a decrease of active TGFβ1 levels was observed in the supernatant (Fig. 3A). This decrease was likely due to some consumption of the BCR-induced expression of TGFβ1 since activation of Smad2/3, a direct cytoplasmic effector of TGFβ signaling, was observed in TGFβ1 expressing samples. Additional exogenous TGFβ1 treatment only resulted in a minor increase in Smad2/3 phosphorylation and, treatment of the B cells with a TGFβ type I receptor inhibitor (SB431542) fully inhibited Smad2/3 phosphorylation in every condition, supporting a self-activating loop as previously described (Fig. S3A and [40]).

Secretion of several other cytokines and factors necessary for regulation of immune cells was detected in CLL B cells supernatants. Low levels of TNFα, IL8 or IL6 were significantly induced upon stimulation (Fig. 3B), while IL4, IL1β, IFNγ, IL2, IL17 and IL13 remained unchanged (Fig. S3B). As shown in Fig. 3C, the regulatory orientation of the culture supernatant was confirmed with an inverted correlation between the secretion of TGFβ1 and those of pro-survival or -inflammatory cytokines. Our results show that CLL cells display a specific secretome and identify immunoregulatory subsets that can further respond to BCR/CD40 stimulation and increase B cell activation.

### Functional impact of secreted IL10 and TGFβ1 on their immune counterparts

To clarify the role of IL10 and TGFβ1 on other immune counterparts, PBMCs from CLL patients were cultured with an anti-IL10 blocking antibody or the TGFβ type I receptor inhibitor (SB431542). Viability of the cells, following 2 days of culture, was not significantly affected up to 10 μg/ml anti-IL10 antibody (left graph) or 5 μM SB431542 (right graph) treatments (Fig. S4A). We confirmed the blocking activity of the anti-IL10 antibody by Western blot. The anti-IL10 and anti-IL10 receptor antibodies efficiently blocked IL10-driven activation of Stat3 at both pSer^727^ and pTyr^705^ (Fig. S4B).

**Fig. 4:**
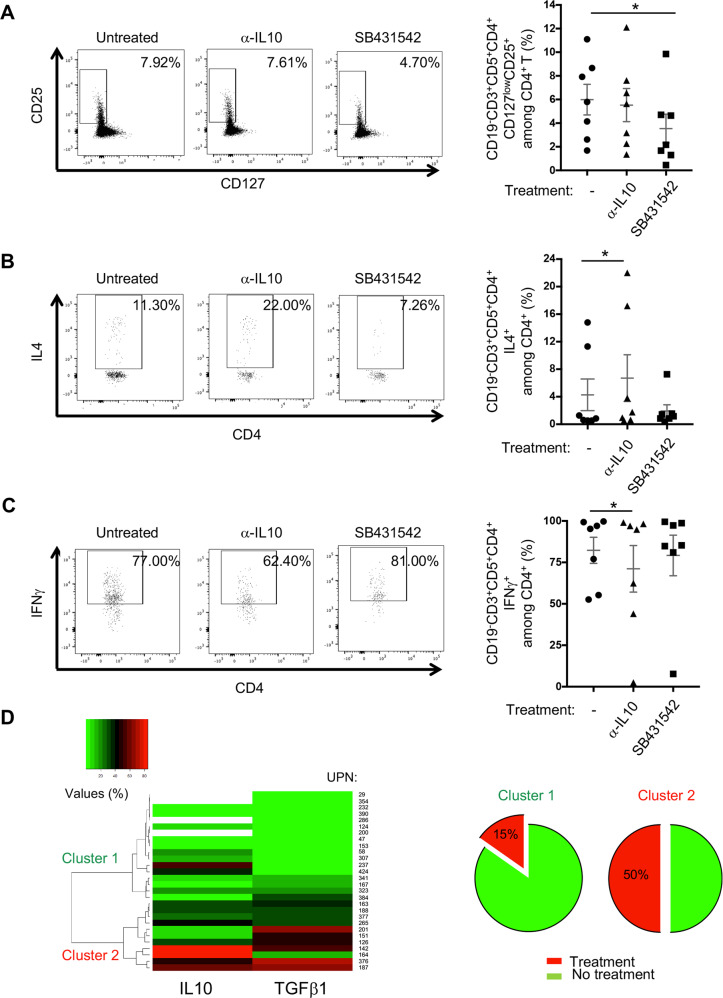
Functional impact of IL10 and TGFβ1 on their immune counterparts. Representative flow cytometry dot plots (UPN 008 and 196) and graphic representation of Treg (CD5^+^ CD4^+^ CD127^low^ CD25^+^) (**A**) Th2 (CD19^−^ CD4^+^ IL4^+^) (**B)** and Th1 (CD19^-^ CD4^+^ IFNγ^+^) (**C**) frequencies in PBMCs (*n* = 7) left untreated (−) or treated with α-IL10 Ab (10 μg/ml) or TGFβRI inhibitor (SB431542, 5 μM) for 2 days. In the representative dot plots, gates indicate positive events with their respective percentages. **D** Unsupervised hierarchical cluster analysis of IL10 and TGFβ1 expression in CLL B cells from a cohort of 28 patients (UPN) allowing the distinction of 2 clusters (1 (green color) & 2 (red color)); immunoregulatory factors frequencies (values (%)) in CLL B cells from each patient are depicted in shades of red (higher) and green (lower). Percentage of treated patients (more than two years) prior to the experiment time in Cluster 1 (15%) and Cluster 2 (50%) are presented as pie (*cf*. Table 1 and Supplementary Table 1 cohort 1).

Given the ex-vivo and in vitro regulatory effects of CLL B cells on immune cells (Fig. 1), we cultured PBMCs from CLL patients for 2 days with or without inhibitors and analyzed Treg, Th2 and Th1 frequencies by flow cytometry (Fig. S4C). Blocking TGFβ signalling decreased Treg frequencies significantly (Fig. 4A), whereas trapping IL10 increased the proportion of IL4-producing T cells and decreased those of IFNγ-ones (Fig. 4B, C). We confirmed a significant decrease of IL10^+^ CLL B cells in anti-IL10-treated PBMCs (*P* = 0.0312) and of TGFβ1^+^ CLL B cells in SB431542-treated PBMCs (*P* = 0.0156), suggesting that both treatments specifically and negatively modulate the expression of their respective intracellular cytokines (Fig. S4D). Also, SB431542 inhibitor did not change significantly the amount of TGFβ1 secreted by CLL B cells (Fig. S4E). These data highlight the critical role of TGFβs in the development and maintenance of the Treg subset and the inhibitory role of IL10 on Th2/Th1 differentiation.

Given that CLL B cells can produce IL10 and TGFβ1 and their functional impact on their immune cells, we examined the clinical relevance of the frequencies of IL10- and TGFβ1-expressing cells. Unsupervised hierarchical cluster analysis of IL10- and TGFβ1-expressing B cells from a first cohort of 28 CLL patients (UPN; Table 1 and Supplementary Table 1) distinguished 2 clusters (Cluster 1 *n* = 17 & Cluster 2 *n* = 11) with a higher proportion of cells expressing both cytokines in group 2 (Fig. 4D). Cluster 2 had a higher number of treated patients (5 out of 10) when compared to cluster 1 (2 out of 13). High proportions of IL10^+^ and TGFβ1^+^ CLL B cells in treated patients, highlighted in this analysis, possibly reflect CLL progression.

### Expression of FOXP3, IL10, TGFβ1 in CLL B cells may constitute a progression indicator

**Fig. 5:**
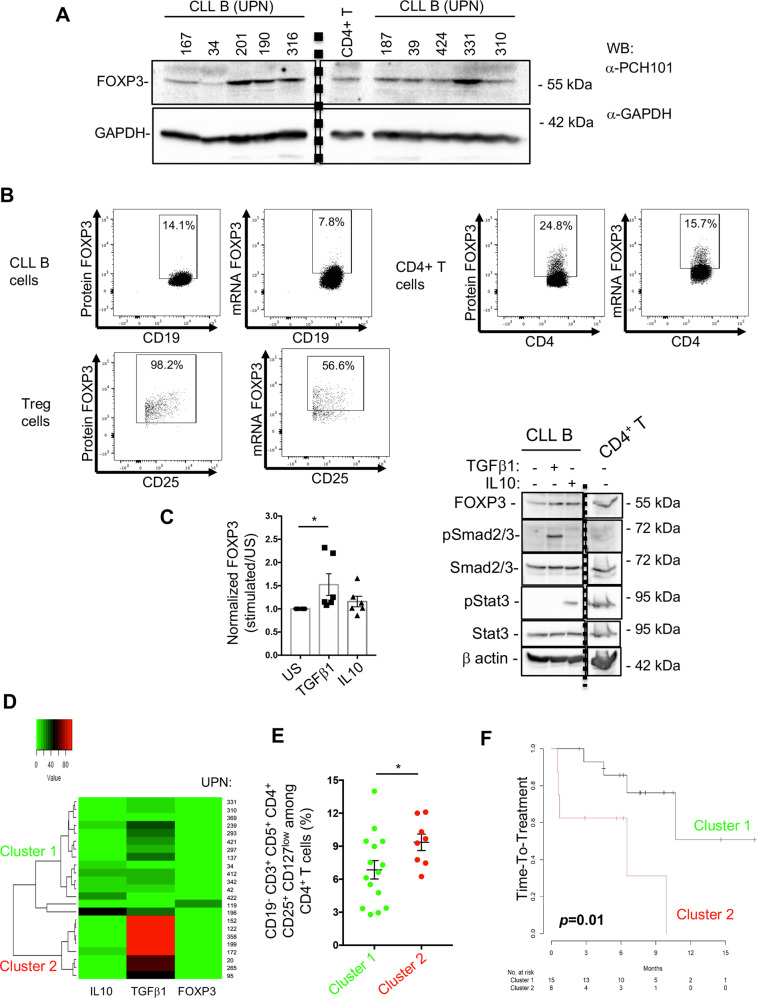
CLL B cells express FOXP3 with IL10 and TGFβ1, which altogether constitutes a progression indicator. **A** Thirty μg of CLL B or CD4^+^T cell lysates from 10 patients (UPN) were analyzed by western blot with the indicated antibodies; GAPDH was used as a loading control. Vertical dashed line separates a cropped membrane split into two parts with the same exposure time (**B**). PBMCs from CLL patients were subjected to RNA flow cytometry protocol, in which CLL B, CD4^+^T and Tregs cells were labeled with dedicated markers and stained for the FOXP3 protein (PCH101) and its RNA (FOXP3 probe). Dot plots depict the targeted cell type and each rectangle shows the positive events with its percentage (UPN 34). **C** CLL B cells were stimulated or not (US) with TGFβ1 or IL10 for 2 days and cell lysates from 6 patients were analyzed by western blot with the indicated antibodies; β-actin was used as loading control. Fold increase of FOXP3 is graphed; Wilcoxon test and *P* = 0.0312. **D** Unsupervised hierarchical cluster analysis of IL10, TGFβ1 and FOXP3 expression in CLL CD19^+^ CD5^+^ CD27^+^ B cells from a cohort of 23 patients (UPN are indicated) determined 2 clusters (1, green and 2, red). Immunoregulatory factors frequencies in CLL B cells are depicted in shades of red (higher) and green (lower). **E** Frequencies of Tregs among PBMCs are graphed and compared between the two clusters; Mann–Whitney test and *P* = 0.0473. **F** According to patients’ clinical annotations (Table 1, Supplementary Table 2 cohort 2), Kaplan–Meier plot shows the time-to-treatment distribution for clusters 1 and 2. Log-rank test was used and *P* value is indicated.

CLL B cells express membrane markers such as CD5 and CD25, which are associated with T and B regulatory cells and regulatory cytokines such as IL10 and TGFβ1. We considered whether CLL B cells may further mirror Tregs by expressing the transcriptional regulator FOXP3. Western blotting with a FOXP3 antibody (clone PCH101) revealed the expression of FOXP3 in purified CLL B cell extracts (Fig. 5A; *n* = 10). CD4^+^ T cells were used as a positive control for FOXP3 expression. We also verified the expression of FOXP3 in CLL B cells using the D6O8R antibody, which recognizes a different FOXP3 epitope than the PCH101 clone. Expression of FOXP3 was enhanced in CD5^+^CD19^+^CD27^+^ cells (Fig. S5A; *n* = 3).

FOXP3 expression was also confirmed by RNA-Flow cytometry on CLL PBMCs (n = 5). We stained CLL B, CD4^+^ and Treg cells for specific extracellular markers and intracellular FOXP3 protein (PCH101) and RNA (FOXP3 probe). While the percentage of FOXP3 positive cells and the median fluorescence intensity were higher in CD4^+^ and Treg cells, CD19^+^CD5^+^ CLL B cells did expressed both FOXP3 RNA and protein (Fig. 5B and S5B). As a mirror of TGFβ− regulation of FOXP3 in Treg cells, TGFβ1-treated CLL B cells showed increased FOXP3 levels while IL10 treatment did not reach significant increase (Fig. 5C), further arguing for different roles between TGFβ1 and IL10. We next examined the clinical relevance of FOXP3^+^ B CLL cells in a second cohort of 23 untreated CLL patients (Table 1 and Supplementary Table 2). We analyzed expression of IL10, TGFβ1 and FOXP3 in CLL B cells (CD19^+^ CD5^+^ CD27^+^) by flow cytometry on thawed PBMC samples. Unsupervised hierarchical clustering of IL10-, TGFβ1- and FOXP3- expression frequencies in CLL B cells revealed the discrimination of two clusters of patients (Fig. 5D; Cluster 1; *n* = 15 and Cluster 2; *n* = 8). A higher proportion of Tregs was found in patients from Cluster 2 when compared to patients in Cluster 1 (Fig. 5E). We assessed time-to-treatment for the second cohort of patients to confirm the clinical significance of these two clusters. A short time-to-treatment was observed in Cluster 2 patients compared to Cluster 1 patients (*p* = 0.01) (Fig. 5F), demonstrating that expression of IL10-, TGFβ1- and FOXP3 in CLL B cells may predict disease progression in CLL patients.

## Discussion

Recent advances in understanding the heterogeneity in risk of progression for CLL patients have established a dysfunction of anti-tumour immune survey. Our study highlights the importance of modulating functions of the clonal B cell expansion, which correlate with disease progression. The analysis of two independent cohorts of CLL B cells and PBMCs from a total of 97 patients demonstrated that CD5^+^CD19^+^CD27^+^ memory CLL B cells produce and secrete IL10 and TGFβ1, as part of the tumour cell secretome. The leukaemic B cell subsets exhibited regulatory functions on their immune counterparts, similar to Bregs. IL10 mainly inhibits Th2/Th1 differentiation while TGFβ induces Tregs. We discovered that FOXP3, a functional marker of Tregs, is also expressed in CLL B cells. Moreover, we found that expression levels of IL10, TGFβ1 and FOXP3 in tumour B cells from untreated CLL B patients were predictive of disease progression.

We validated the regulatory properties of the leukaemic B cell populations in PBMCs and showed a significant drop of in the number of monocytes, as well as an expansion of CD4^+^ Tregs. Co-culture experiments with autologous T cells confirmed that leukaemic B cells drive the expansion of CD4^+^CD25^+^CD127^low^FOXP3^+^ Tregs and the reduction of effector T cells expressing TNF-α and IFNγ. The T cell subsets are involved in tumour clearance and anti-pathogens response [20, 23, 41]. Furthermore, the changes observed in cellular immune composition were cellular ratio-dependent. Indeed, the CD4^+^T/CLL B cells ratio used for the co-culture experiment were reflected the cellular bias observed in vivo for CLL patients [4, 42]. These findings refer to hallmarks of functional Bregs and are in line with the acquisition of regulatory properties for CLL cells contributing to the disruption of immune survey mechanisms and tumor escape.

IL10 and TGFβ1 are key regulators of immune homeostasis. Several reports have implicated IL10-producing CLL B cells (B10, [24, 43]) in the dysfunction of the T cell compartment accompanying disease progression [3, 21, 24, 25]. CLL B cells are also described as TGFβ1-producing cells [3, 40]. Both immunomodulatory factors are important contributors to the peripheral conversion of CD4^+^ T cells into FOXP3^+^ induced Tregs (iTregs) during an active immune response [3, 31, 44]. Interestingly, iTregs also produce both IL10 and TGFβ1, are found in solid tumour infiltrates and are indicative of a poor anti-tumour response during cancer progression [45]. In accordance with this regulatory B cell role, we demonstrated in CLL that TGFβ contributes to the conversion of helper T cells into Tregs, without affecting directly the Th1 or Th2 frequencies. In the context of a Th2/Th1 imbalance in CLL cases [2, 13], IL10 had an effect on the differentiation of Th1/Th2 cells. Given the low levels of IL10 secreted in CLL B cells, future dose-response experiments should clarify whether a threshold of IL10 secretion is mandatory to suppress the differentiation. We demonstrated the heterogeneous expression of IL10 and TFGβ in B cells from CLL patients. We highlighted the presence of various CLL B cell subsets expressing either only one or both cytokines. These findings strengthen the notion of the heterogenous immune modulating functions of the clonal B cell expansion.

Phenotypic assessment of the regulatory populations in autoimmune diseases led to the description of various subtypes with IL10- or TGFβ1-dependent suppressive mechanisms [17, 19]. An initial study on CLL characterized a B10-like CD19^+^CD24^hi^CD27^+^ regulatory subset [21]. CD27 marker was also found differentially expressed between IL10^+^ and IL10^-^ cells in our experiments and the CD5^+^CD19^+^CD27^+^ subpopulation contributed to the expression of IL10. Several other subtypes, such as immature CD19^+^CD24^hi^CD38^hi^, CD1d^+^CD19^+^CD38^+^IgM^+^CD147^+^GrB^+^ or CD11b^+^CD19^+^ cells, have also been described as IL10 competent cells [20, 21, 23, 36, 46]. CD11b^+^ and GrB^+^ cells did not represent a significant proportion of CLL B cells (data not shown) and GrB expression was not significantly induced upon IL21/CD40L triggering as compared to healthy controls. A study identified the CD19^+^CD24^+^CD38^hi^ CLL subset as IL10 and TGFβ1 high producer cells responsible for transforming naïve CD4^+^ T cells into Tregs, while IL10 production in IGHV mutated CLL cells was correlated with anergy [3, 25]. Other studies identified CTLA4-induced regulatory B cells as TGFβ1 and IDO producers [31]. TGFβ1 is mandatory for Tregs induction but also for CD8^+^ T cell anergy and these immuno-modulations have been documented in CLL [12, 47, 48]. In our CLL cohort, CD5^+^CD19^+^CD27^+^ cells represented the discriminant phenotypic features of IL10^+*vs*-^ and TGFβ1^+*vs*-^ subsets, which also resembled CD19^+^CD81^+^CD27^+^CD25^+^PD-L1^hi^ tumor evoked Bregs producing both IL10 and TGFβ1 in sarcomas [49]. As we observed with the decrease of CD5 expression in BCR/CD40 stimulated cells, many surface markers used to identify Bregs are up- or down-regulated during immune activation [17]. Overall, the heterogeneous features of CLL regulatory B cells cannot be limited to one specific phenotype, and this heterogeneity is due to the induction and activation by surrounding cells.

Tregs are key players in the maintenance of immune tolerance, and their expansion is regulated during this process. Also, a comprehensive role in tolerance has been attributed to regulatory B cells which exhibit a number of common features with Tregs, among which IL10 or TGFβ1 production. Loss of function or number decrease in Breg cells result in autoimmune diseases [19]. Interestingly, we demonstrated that CLL B cells, not only express IL10 and TGFβ1 but also the transcription factor FOXP3. Using various techniques, we demonstrated the expression of both FOXP3 mRNA and protein in purified CLL B cells after exclusion of any Tregs or other immune cells, and using FOXP3-positive cells such as Tregs and CD4^+^ T cells as a control. FOXP3 expression has been described in tumour infiltrating Tregs in epithelial solid tumours such as pancreatic adenocarcinoma, in glioma cells and in Bregs present in systemic lupus erythematosus (SLE) [50–52]. Anti-tumour and oncogenic properties have been attributed to FOXP3 depending on the cellular context and interacting partners [53]. For example, FOXP3 interaction with NFAT or NFκB regulates anti-tumour immunity [54, 55]. TGFβ1 and TGFβ2 have been implicated in FOXP3 induction in peripheral Tregs and adenocarcinoma expansion [56]. Moreover, FOXP3 and TGFβ1 are concomitant intermediates in Tregs/Th17 homeostasis. CLL B cells, also expressed CD25, another key marker and regulator of Tregs, which has been observed in IL10^+^ mature Breg cells with antigen specific suppressive functions in SLE patients [52]. Furthermore, FOXP3 associates with STAT3, which is constitutively phosphorylated at S^727^ in CLL, to promote the expression of IL10 in a subset of Tregs. FOXP3 expression is also regulated by both CD5 and TGFβ1 in this subset of Tregs [57]. Interestingly, we found that TGFβ1-treatment also leads to higher expression of FOXP3 in CLL B cells. Whether some coordinated regulation of FOXP3, TGFβ1, CD5 and CD25 expressions may occur in CLL B cell subtypes remains to be determined.

Recent studies have highlighted the integrative evaluation of various immune subsets and the production of cytokines and soluble factors as better predictors of a dysfunctional immune survey [58]. Such combinatorial analysis of IL10 and TNFα expression in CD19^+^CD24^hi^CD38^hi^ Bregs proved better efficacy than individual evaluation during renal allograft rejection [59]. Similarly, computing the expression of various cytokines with a polyfunctional index delineated a heterogeneous response in infectious disease [60]. Our work showed that using an unsupervised hierarchical cluster analysis of IL10, TGFβ1 and FOXP3 underscored the heterogeneous profile of the patients and may predict disease progression. Our unsupervised hierarchical cluster analysis differentiated two clusters of patients based on the proportion of CLL B cells expressing IL10, and TGFβ1. In a first cohort, our analysis on purified CLL B cells showed a differential association with CLL progression between the two clusters of patients. The relevance of this analysis was further confirmed in a second cohort with PBMCs of untreated patients for which the need of treatment was considered. Therefore, the characterization and quantification of the regulatory-competent cells should improve the prediction for the heterogeneous course and survival outcome of CLL patients. In line with this evaluation, the differential proportion of Tregs observed in the two clusters may also be relevant. Clustering IL10, TGFβ1 and FOXP3 may also provide a better understanding of the contribution of the regulatory subsets to the systemic immunodeficiency and lack of tumour clearance observed in CLL patients.

All these data argue for a comprehensive regulation of the three immunomodulatory factors with a differential expression among CLL patients at various stages of the disease.

## Supplementary information

Manuscript Supplementary information AM et al CLR accepted 15 02 23

## Data Availability

The main data supporting the results in this study are available within the article and its Supplementary Information. All data generated in this study are available from the corresponding authors upon reasonable request.
