# Supplementary material for: Immuno-regulatory malignant B cells contribute to Chronic Lymphocytic Leukemia progression
Source: Cancer Gene Ther. 2023 Mar 28;30(7):1018–28. doi: 10.1038/s41417-023-00602-5 (PMC10353928; doi:10.1038/s41417-023-00602-5)
Supplement: Supplementary file 1 — Manuscript Supplementary information AM et al CLR accepted 15 02 23 [file 41417_2023_602_MOESM1_ESM.pdf]

Supplementary Information

Immuno-regulatory malignant B cells contribute to Chronic Lymphocytic Leukemia progression

Arsène Mékinian [1, 2], Anne Quinquenel [§, 1, 2], Koceïla Ait Belkacem [§, 1, 2], Feriel Kanoun [1, 2], Elisabetta Dondi [1, 2], Emilie Franck [1, 2], Marouane Boubaya [3], Maïssa Mhibik [1, 2], Fanny Baran-Marszak [1, 2, 4], Rémi Letestu [1, 2, 4], Florence Ajchenbaum-Cymbalista [1, 2, 4], Vincent Lévy [3, 5], Nadine Varin-Blank * [1, 2] and Christine Le Roy * [1, 2].

[1] INSERM, U978, Bobigny, France

[2] Université Paris 13 dite « Sorbonne Paris Nord », UFR SMBH, Labex INFLAMEX, Bobigny, France

[3] URC, APHP, Hôpital Avicenne, Bobigny, France

[4] Service d'Hématologie Biologique, APHP, Hôpital Avicenne, Bobigny, France

[5] CRC, APHP, Hôpital Avicenne, Bobigny, France

These authors contributed equally to this work:

[§] Co-second authors and

* Co-senior and -corresponding authors, UMR InsermU978 / Université Paris 13 dite « Sorbonne Paris Nord », UFR SMBH, 74 Rue Marcel Cachin, 93017 Bobigny, France; Emails: nadine.varin@inserm.fr and christine.le-roy@inserm.fr

Competing Interests statement: none

Keywords: IL10, TGFβ1, FOXP3, Regulatory B cells, CLL progression

Running title: Immuno-regulatory functions in CLL progression

Supplementary information: Methods

Western blotting

CLL B cells purified from individuals and $CD4^+$ T cells pooled from 10 CLL samples were lysed in RIPA buffer (25 mM Tris-HCl, pH 7.4, 150 mM NaCl, 1 mM EDTA, 1% NP40, 1% sodium deoxycholate, 0.1% SDS) supplemented with protease inhibitors (0.2 mM Na_3VO_4, 25 mM NaF, 20 μg/ml Aprotinin, 10 μg/ml Leupeptin, 10 μg/ml Pepstatin and 1 mM PMSF). Negatively selected and sorted B cells, U2OS, as well as MOCK- or FOXP3-transfected HEK293T cells were lysed in 1% NP-40 lysis buffer (50 mM Tris-HCl pH 7.4, 150 mM NaCl, 1 mM EDTA, 1% NP-40, 10% Glycerol with protease inhibitors). IL10 or TGFβ1 stimulated, α-IL10, α-IL10 receptor or SB431542 treated CLL B cells were lysed in 2% NP-40 lysis buffer or with RIPA buffer containing anti-proteases.

Flow cytometry

Isolated PBMCs were cultured or not for 2 days, stained for viability (FVS-510, Becton Dickinson) for 10 min at 4°C according to manufactures' instructions, washed, labelled with the indicated antibodies in 2% FCS/PBS for 1h at 4°C and washed. Monocytes, B and T cell subsets were analyzed on FACSCanto II driven by the BD FACSDIVA™ software and data compiled with the FlowJo™ software (BD Biosciences).

Following 3 days of co-culture, cells were labelled with antibodies for 30 min at 4°C, washed in 2% FCS/PBS, fixed with 2% PFA/PBS for 20 min at 4°C, permeabilized with 0.5% saponin, 1% BSA in PBS for 30 min at room temperature, stained for FOXP3 in permeabilization buffer for 1h at room temperature and analyzed on the FACSCanto II (BD). A similar protocol was

used for IL10 and TGFβ1 intracellular staining on 48h-cultured PBMCs and on 72h-anti-IgM/CD40L stimulated or not B cells.

Cytoplasmic TNFα and IFNγ in $CD4^+$ T cells was analyzed after treatment with PIB. Briefly, after staining with Lifedead V500 (Ebiosciences) for 10 min at 4°C, cells were labelled with antibodies for 20 min at 4°C, fixed and permeabilized with Cytofix/Cytoperm buffer (BD Biosciences) following the manufacturer's protocol and then incubated with anti-TNFα and -IFNγ or control isotype for 1h before processing on FACS Canto II (BD).

The functional impact of anti-IL10 or TGFβRI inhibitor on T cell subsets (IL4 and IFNγ) was analyzed on PBMCs cultured or not for 2 days, treated with PIB, stained for viability (FVS440UV BD) before extracellular (20 min at 4°C) and intracellular staining (1h at 4°C) using the Cytofix/Cytoperm kit (BD Biosciences). Cell subsets were analyzed on a Symphony™ A3 SORP analyzer (Becton Dickinson).

Intracellular staining for IL10, TGFβ1 and FOXP3 was performed on 48h-cultured PBMCs (cohort #2) treated with PIB, stained for viability (BD Horizon Fixable Viability Stain 440UV) for 10 min at 4°C and labelled for 30 min at 4°C with a mix of antibodies diluted in PBS supplemented with 2% FCS and 10% BD Horizon Brilliant Stain Buffer Plus. Using the FOXP3 Transcription Factor staining buffer set (eBioscience, ThermoFisher Scientific) and according to manufacturer's protocol, cells were fixed for 45 min at RT, permeabilized for 10 min with 5% Fc Block™ (BD) and then incubated with a mix of antibodies targeting both cytokines and the transcription factor for 45 min or with isotypic controls. Samples were analyzed on the Symphony™ A3 SORP analyzer (Becton Dickinson).

For detection of RNA targets, B cells or PBMCs were processed according to the manufacturer's instructions (PrimeFlow RNA assay, Invitrogen, ThermoFisher Scientific) using target-specific probe sets. Briefly, cells were treated with PIB, extracellularly labelled with a mix of antibodies for 30 min at 4°C and intracellularly stained with or without the RNA

probes targeting IL10 and TGFβ1. Samples were analyzed on Canto II flow cytometer (BD).
For simultaneous FOXP3 mRNA and protein detection, thawed PBMCs ($2x10^6$) were cultured for 2 days, treated with PIB, stained for dead cells (BD Horizon Fixable Viability Stain 440UV) for 10 min at 4°C and extracellularly labelled for 30 min at 4°C with a mix of antibodies diluted in PBS supplemented with 2% FCS and 10% BD Horizon Brilliant Stain Buffer Plus. Cells were then fixed, incubated with human BD Fc Block™ for 10 min at 4°C before being intracellularly stained for FOXP3 or an irrelevant antibody for 45 min at 4°C in 1X PrimeFlow RNA permeabilization buffer. After a second step of fixation, cells were incubated with an RNA probe targeting FOXP3 for 2 h at 40°C, the signal was amplified by sequential steps and CLL B, $CD4^+$ T and Treg were analyzed on the Symphony™ A3 SORP analyzer before processing with FlowJo.

Supplementary Figures and Tables

Supplementary Figure 1: CLL cells undertake a regulatory crosstalk with their immune counterparts

(**A**) Representative gating strategy for the selection of monocytes (CD14 *vs* CD16) and of single viable CLL B (CD5 *vs* CD19) and T cells (CD8 *vs* CD4) (UPN 119). (**B**) Representative histogram stagger offset of the CD19 MFI among $CD4^+$ and B cells co-cultures depicting the various ratios of autologous purified cell types (1st peak: $CD4^+$ T - $CD19^-$; and 2nd peak: CLL B - $CD19^+$) (UPN 112). (**C**) Representative dot plots showing the frequencies of TNF-α (top series) and IFN-γ (bottom series) in unstimulated (NS) and stimulated (S) $CD4^+$ T cells co-cultured at the indicated ratios with CLL B cells (UPN 112); gates were determined based on the respective isotype antibodies.

Supplementary Figure 2: Specific subsets of CLL B cells express IL10 together with TGFβ1.

(**A**) Representative gating strategy for selection of single and viable CLL B cells ($CD5^+$ *vs* $CD19^+$) expressing IL10 or TGFβ1; numbers indicate the frequencies of these populations obtained after flow cytometry analysis and gates were determined based on the respective negative control staining (UPN 239). (**B**) Representative histogram overlays of IL10 (red) or TGFβ1 (blue) mRNA expression in CLL B cells from UPN 369 using RNA flow methodology; the RPL13A probe (green) was used as a positive control. Percentage of IL10- or TGFβ1-mRNA expressing CLL B cells among $CD19^+CD5^+$ cells obtained by RNA flow cytometry; dotted lines link individual patient samples. Quantification of CLL B cells co-expressing IL10 and TGFβ1 mRNA (n=5). (**C**) Representative dot plots of purified unstimulated or CD40L-α-IgM-stimulated CLL B cells labelled with α-IL10 or α-TGFβ1 antibody and their respective

isotype controls (UPN 258). (**D**) Purified B cells were cultured for 3 days, stimulated with anti-IgM/CD40L, labelled with the indicated membrane markers and stained for IL10 (top) or TGFβ1 (bottom). MFI ratio of CD5, 19, 27, 24, 25, 27 and 38 between IL10$^{+/-}$ (n=16) and TGF-β1$^{+/-}$ (n=15) cells are graphed (*cf.* Supplementary Table 4). Wilcoxon signed-rank test * P≤0.05, ** P≤0.01, **** P≤0.0001. (**E**) Frequencies of Granzyme B^{+} in CLL or healthy control B cells were evaluated upon CD40L/anti-IgM or CD40L/IL-21 stimulation as a control by flow cytometry. Mann Whitney test with * P≤0.05 and *ns*, not significant.

Supplementary Figure 3: CLL B cells are sensitive to TGFβ1 stimulation and secrete BCR-independent cytokines.

(**A**) CLL B cells were treated with (+) or without (-) with exogenous TGFβ1 (5 ng/ml) in the presence of the indicated concentrations of SB 431542. After two days, total cell lysates were analyzed by western blot with the indicated antibodies (UPN 334). Quantification of both phospho-Smad2/3 normalized to Smad2/3 was graphed (n=3). (**B**) Quantities of the indicated cytokines secreted in the culture supernatant of purified CD5^{+}CD19^{+} cells, stimulated (+) or not (-) with anti-IgM/CD40L and analyzed by “multi-ELISA” assay. Wilcoxon matched-paired signed rank test, *ns*, not significant P>0.05.

Supplementary Figure 4: Functional impact of IL10 and TGFβ1 on their immune counterparts.

A) Graphs depict viable PBMCs number (left, n=4) or percentage (right, n=6)) upon 2 days-treatment with increasing concentrations of α-IL10 or SB 431542, respectively. (**B**) CLL B cells were treated with (+) or without (-) with exogenous IL10 (40 ng/ml) in presence (+) or absence (-) of inhibitors targeting the soluble cytokine (anti-IL10; 10 μg/ml) or blocking the binding to its cognate receptor (anti-R-IL10; 2.5 μg/ml). After two days, total cell lysates were

hybridized with the indicated antibodies (UPN 358). Quantification of both phospho-Stat3 normalized to Stat3 was graphed (n=6); Wilcoxon test * P= 0.0312. (**C**) Representative gating strategy used in flow cytometry analysis to target CD4$^+$T cells from 48h cultured PBMCs (UPN 196). (**D**) Graphs showing the percentages of IL10$^+$ and TGFβ1$^+$ CLL B cells among PBMC after treatments or not (-) with anti-IL10- or SB 431542 from 7 CLL samples; Wilcoxon test * P= 0.0469 and P= 0.0156 respectively). (**E**) Graph depicting secreted TGFβ1 in supernatants from untreated (-) or SB 431542-treated CLL B cells (n=7).

Supplementary Figure 5: FOXP3 is expressed in CD19$^+$CD5$^+$CD27$^+$CLL B cells.

(**A**) Cell sorting gating strategy of B cells negatively selected for CD19$^+$ CD3$^-$ CD5$^+$ CD27$^+$; numbers are percentages of cells for each dot plot. Western blot analysis of FOXP3 expression using the D608R antibody on purified as well as purified and subsequently sorted CLL B cells. U2OS and HEK293T cells transfected with FOXP3 or Mock expressing vectors were used as controls. Vertical dashed line separates a cropped membrane split into two parts with the same exposure time (**B**) Gating strategy for viable PBMCs subjected to RNA Flow cytometry approach shown in Figure 5B (UPN 34). (**C**) Gating strategy for viable CLL B cells stained for IL10, TGFβ1 and FOXP3 (UPN 265).

Supplementary Table 1: Features of CLL patients in the cohort #1 (n=28)

Supplementary Table 2: Features of CLL patients in the cohort #2 (n=23)

Supplementary Table 3: Antibodies and probes used in this study.

Supplementary Table 4: MFI of the CD markers used for Figures 2D and S2D

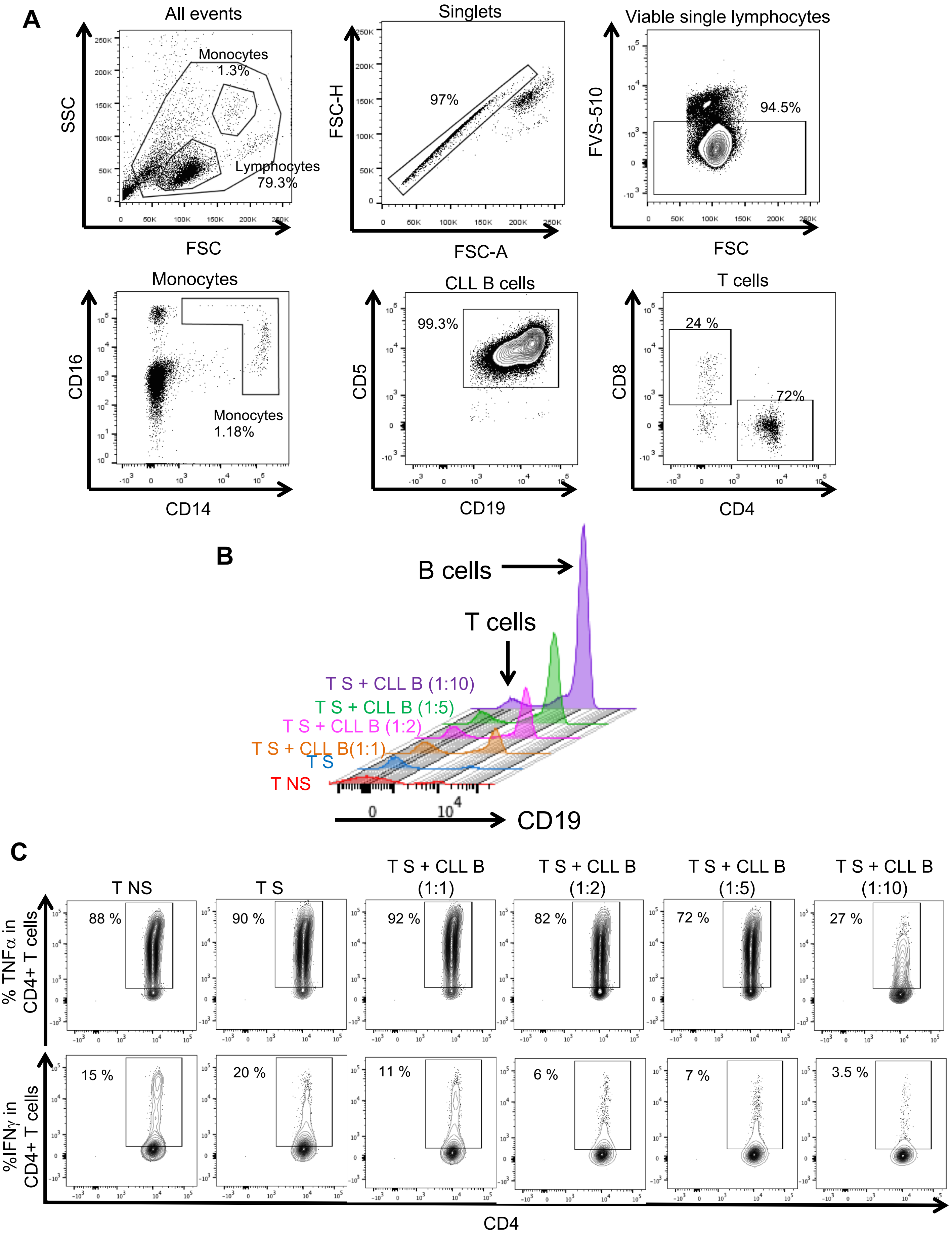
A
All events
Monocytes
1.3%
Lymphocytes
79.3%
SSC
FSC
Singlets
97%
FSC-H
FSC-A
Viable single lymphocytes
94.5%
FVS-510
FSC
Monocytes
Monocytes
1.18%
CD16
CD14
CLL B cells
99.3%
CD5
CD19
T cells
24 %
72%
CD8
CD4
B
B cells
T cells
T S + CLL B (1:10)
T S + CLL B (1:5)
T S + CLL B (1:2)
T S + CLL B(1:1)
T S
T NS
CD19
C
T NS
T S
T S + CLL B (1:1)
T S + CLL B (1:2)
T S + CLL B (1:5)
T S + CLL B (1:10)
% TNFα in CD4+ T cells
88 %
90 %
92 %
82 %
72 %
27 %
%IFNγ in CD4+ T cells
15 %
20 %
11 %
6 %
7 %
3.5 %
CD4

FIGURE S1

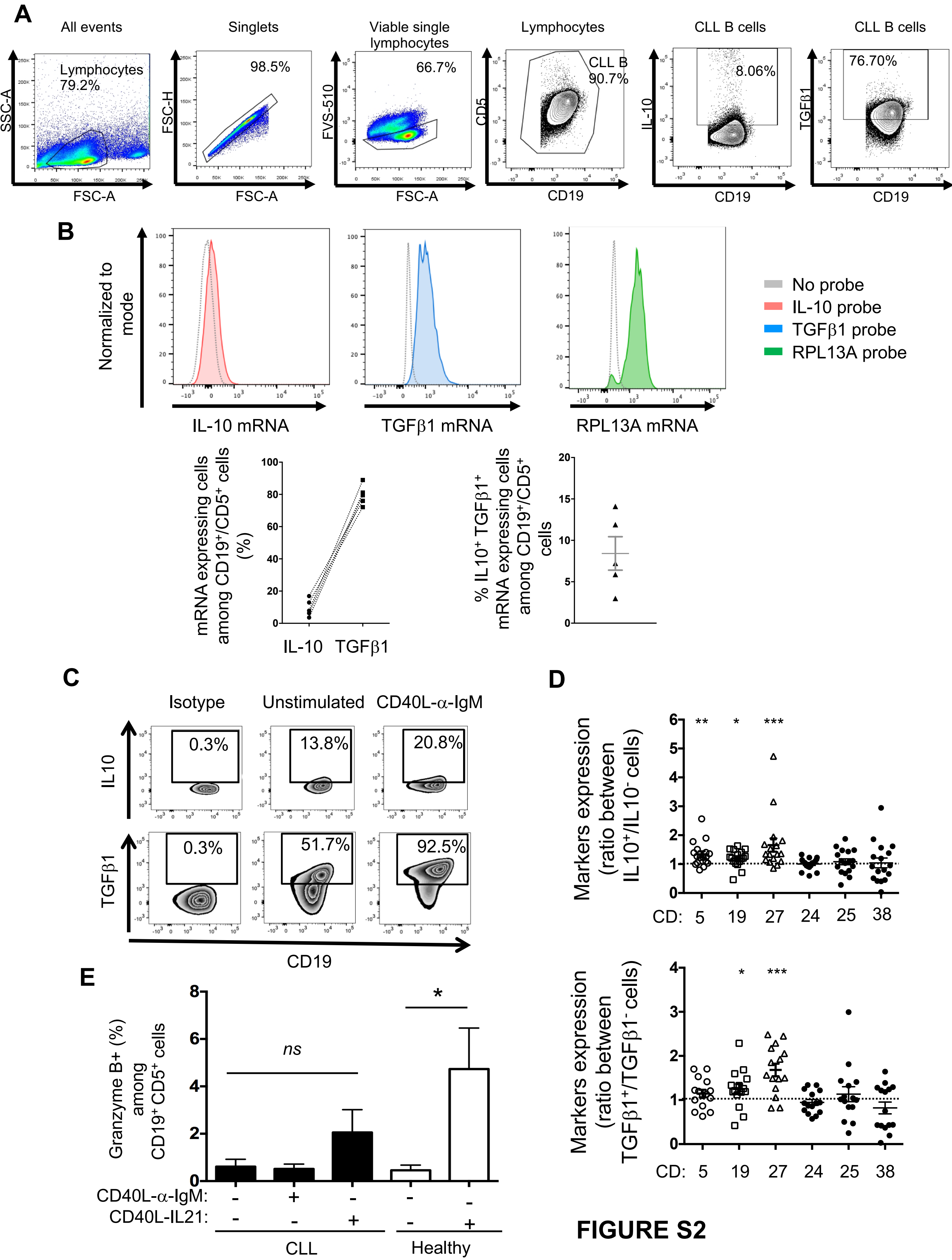
A
All events
Singlets
Viable single lymphocytes
Lymphocytes
CLL B cells
CLL B cells
Lymphocytes 79.2%
98.5%
66.7%
CLL B 90.7%
8.06%
76.70%
SSC-A
FSC-H
FVS-510
CD5
IL-10
TGFβ1
FSC-A
CD19
B
Normalized to mode
IL-10 mRNA
TGFβ1 mRNA
RPL13A mRNA
No probe
IL-10 probe
TGFβ1 probe
RPL13A probe
mRNA expressing cells among CD19+/CD5+ cells (%)
IL-10
% IL10+ TGFβ1+ mRNA expressing cells among CD19+/CD5+ cells
C
Isotype
Unstimulated
CD40L-α-IgM
IL10
0.3%
13.8%
20.8%
51.7%
92.5%
D
Markers expression (ratio between IL10+/IL10- cells)
Markers expression (ratio between TGFβ1+/TGFβ1- cells)
CD: 5 19 27 24 25 38
**
*
***
E
Granzyme B+ (%) among CD19+ CD5+ cells
ns
CD40L-α-IgM:
CD40L-IL21:
CLL
Healthy

FIGURE S2

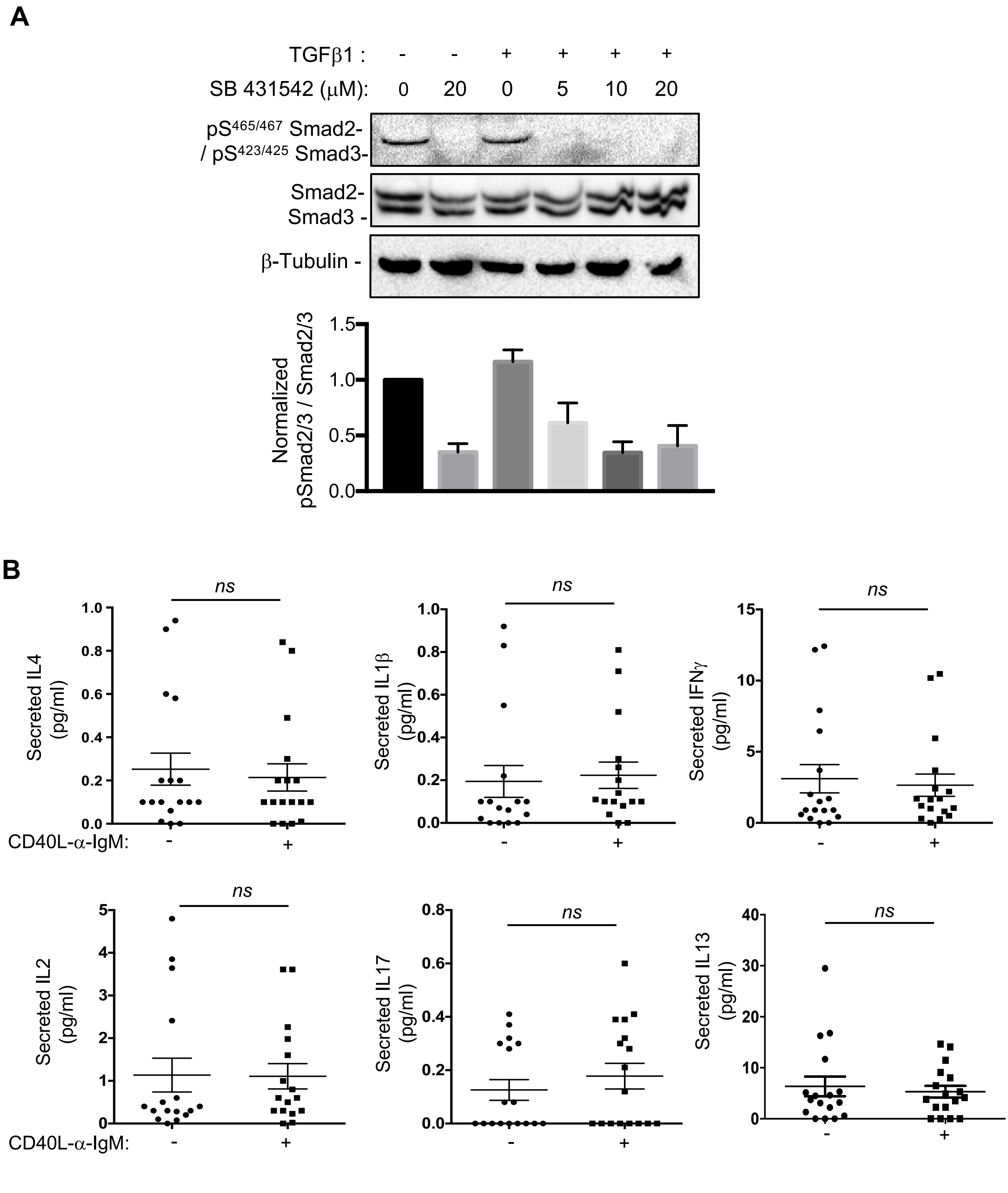
A
TGFβ1 :
-
-
+
+
+
+
SB 431542 (μM):
0
20
0
5
10
20
pS465/467 Smad2-
/ pS423/425 Smad3-
Smad2-
Smad3 -
β-Tubulin -
Normalized
pSmad2/3 / Smad2/3
1.5
1.0
0.5
0.0
B
ns
Secreted IL4
(pg/ml)
Secreted IL1β
(pg/ml)
Secreted IFNγ
(pg/ml)
Secreted IL2
(pg/ml)
Secreted IL17
(pg/ml)
Secreted IL13
(pg/ml)
CD40L-α-IgM:
-
+

FIGURE S3

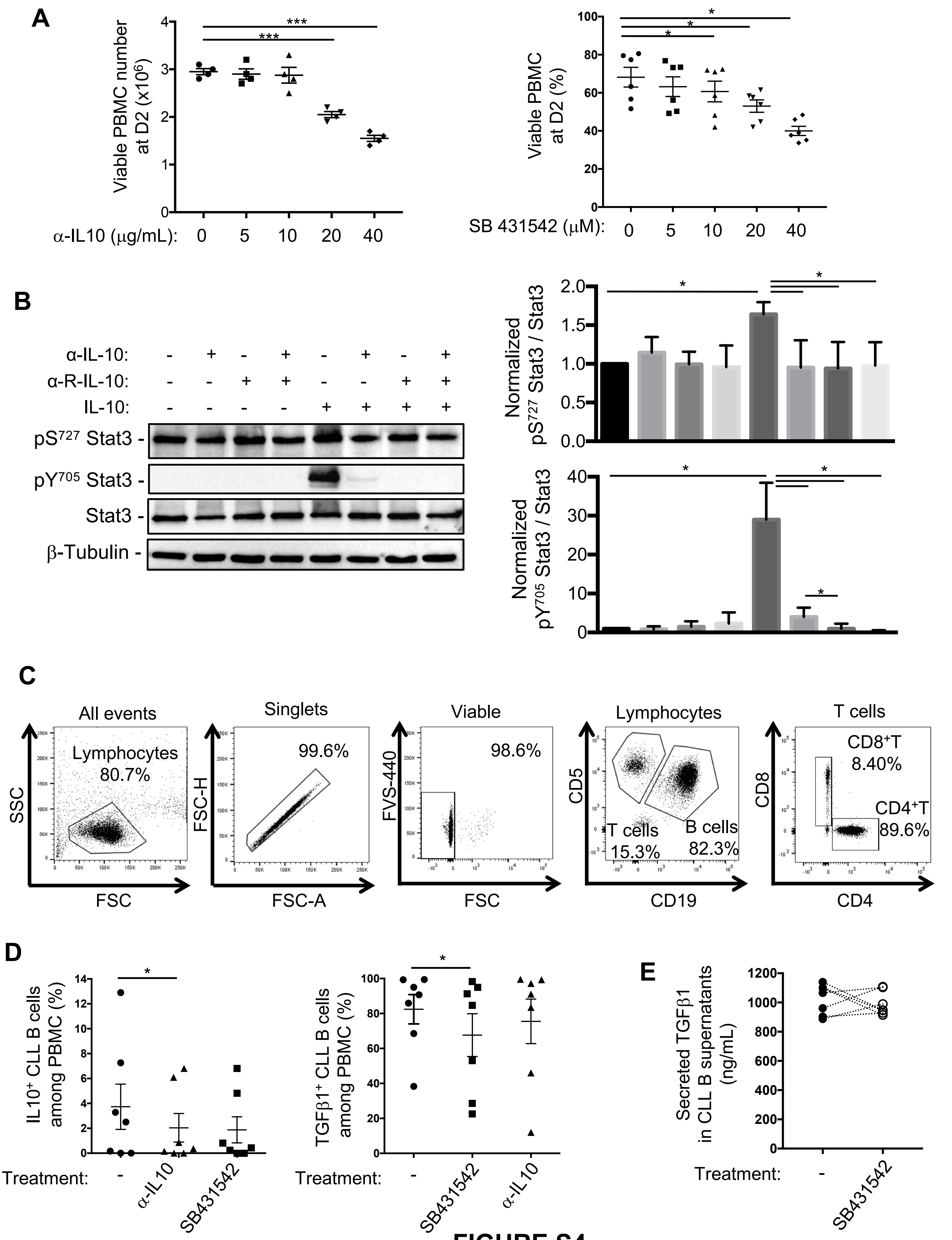
A
Viable PBMC number at D2 (x10^6)
α-IL10 (μg/mL): 0 5 10 20 40
***
Viable PBMC at D2 (%)
SB 431542 (μM): 0 5 10 20 40
*
B
α-IL-10:
α-R-IL-10:
IL-10:
pS727 Stat3 -
pY705 Stat3 -
Stat3 -
β-Tubulin -
Normalized pS727 Stat3 / Stat3
Normalized pY705 Stat3 / Stat3
C
All events
Lymphocytes 80.7%
SSC
FSC
Singlets
99.6%
FSC-H
FSC-A
Viable
98.6%
FVS-440
FSC
Lymphocytes
CD5
CD19
T cells 15.3%
B cells 82.3%
T cells
CD8+T 8.40%
CD4+T 89.6%
CD8
CD4
D
IL10+ CLL B cells among PBMC (%)
Treatment: - α-IL10 SB431542
TGFβ1+ CLL B cells among PBMC (%)
Treatment: - SB431542 α-IL10
E
Secreted TGFβ1 in CLL B supernatants (ng/mL)
Treatment: - SB431542

FIGURE S4

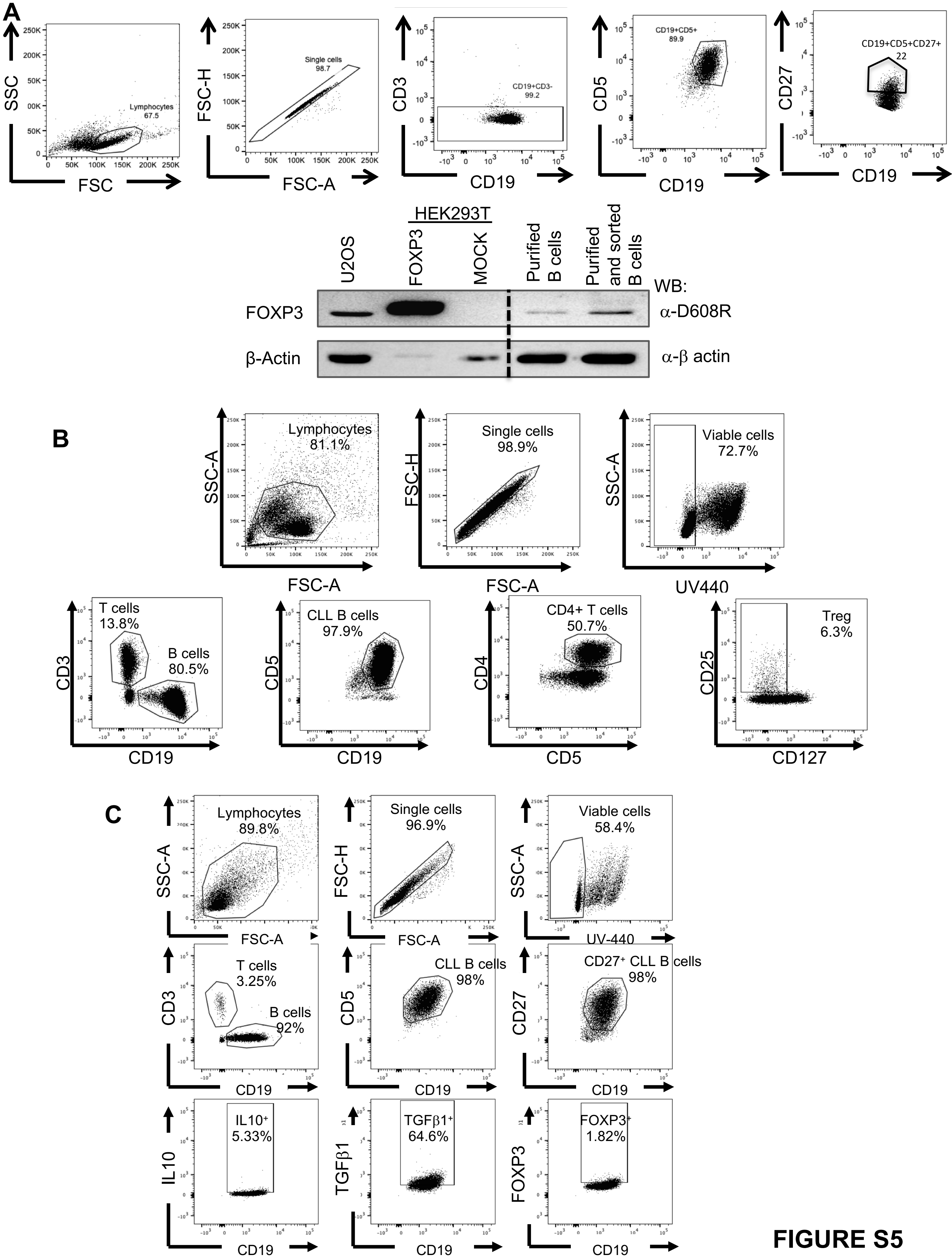
A
SSC
FSC
Lymphocytes 67.5
FSC-H
FSC-A
Single cells 98.7
CD3
CD19
CD19+CD3- 99.2
CD5
CD19+CD5+ 89.9
CD27
CD19+CD5+CD27+ 22
HEK293T
U2OS
FOXP3
MOCK
Purified B cells
Purified and sorted B cells
WB:
FOXP3
α-D608R
β-Actin
α-β actin
B
SSC-A
FSC-A
Lymphocytes 81.1%
FSC-H
Single cells 98.9%
Viable cells 72.7%
UV440
T cells 13.8%
B cells 80.5%
CLL B cells 97.9%
CD4+ T cells 50.7%
CD4
Treg 6.3%
CD25
CD127
C
Lymphocytes 89.8%
Single cells 96.9%
Viable cells 58.4%
UV-440
T cells 3.25%
B cells 92%
CLL B cells 98%
CD27⁺ CLL B cells 98%
IL10⁺ 5.33%
IL10
TGFβ1⁺ 64.6%
TGFβ1
FOXP3⁺ 1.82%
FOXP3

FIGURE S5

Supplementary Table 1: Features of CLL patients in the cohort #1 (n=28)

Cluster #	UPN	Age[A]	Sex[B]	Binet stage[C]	IGVH status[D]	Need of treatment[E]
1	29	56	M	A	UM	0
	354	65	M	B	UM	NA
	232	66	M	B	M	1
	390	55	M	A	UM	0
	286	64	M	A	M	0
	124	48	M	A	M	0
	200	55	M	A	M	NA
	47	75	M	A	M	0
	153	85	F	A	M	0
	58	71	F	A	M	NA
	307	56	F	A	M	0
	237	59	M	A	ND	0
	424	61	M	B	UM	NA
	341	45	M	C	UM	0
	167	59	F	B	UM	0
	323	68	F	A	M	0
	384	58	M	C	UM	1
2	163	63	M	A	UM	0
	188	53	M	A	M	1
	377	78	F	A	M	0
	365	72	F	A	UM	0
	201	49	M	C	UM	1
	151	74	F	C	UM	1
	126	53	M	A	M	NA
	142	60	F	C	UM	1
	164	70	F	A	UM	0
	376	48	M	C	UM	1
	187	64	M	A	M	0

Footnotes

A. Patient's age at the experiment time (year)
B. Female (F) and Male (F)
C. Stage A, B or C at the experiment time
D. Unmutated (UM) or Mutated (M) IGHV status
E. Need of treatment (1) or not (0) prior to the experiment time (more than 2 years)

ND: Not Determined; NA: Not available

Green: patients'cluster #1; Red: patients' cluster #2 (*cf.* Figure 4D)

Supplementary Table 2: Features of CLL patients in the cohort #2 (n=23)

Cluster #	UPN	Age[A]	Sex[B]	Binet stage[C]	IGVH status[D]	Need of treatment[E]
1	331	76	F	A	M	0
	310	67	F	B	UM	1
	369	62	F	A	M	0
	239	76	M	A	M	0
	293	65	M	A	M	0
	421	80	M	A	M	0
	297	68	F	A	M	0
	137	66	F	A	M	0
	34	92	F	A	M	0
	412	74	M	B	M	1
	342	40	M	A	UM	0
	42	92	F	C	UM	1
	422	57	M	A	M	0
	119	62	M	B	UM	1
	196	85	M	B	UM	1
2	152	75	F	B	M	1
	122	59	M	C	UM	1
	358	62	M	C	UM	1
	199	42	M	C	UM	1
	172	70	M	B	UM	1
	20	53	M	A	M	0
	265	54	M	A	UM	0
	95	62	M	A	UM	0

Footnotes

A. Patient's age at the experiment time (year)
B. Female (F) and Male (F)
C. Stage A, B or C at the experiment time
D. Unmutated (UM) or Mutated (M) IGHV status
E. Need of treatment (1) or not (0) after the experiment time

Green: patients'cluster #1; Red: patients' cluster #2 (*cf.* Figure 5D)

Supplementary Table 3: Antibodies and probes used in this study.

Application	Antibody name	Clone #	Catalog #	Fluorochrome	Company
**Cell sorting**	CD19	LT19	130-113-170	PE-Vio770	Miltenyi Biotec, Paris, France
	CD3	REA613	130-113-141	PerCP-Vio700	Miltenyi Biotec
	CD5	UCHT2	130-119-852	APC-Vio770	Miltenyi Biotec
	CD27	M-T271	130-113-633	VioBlue	Miltenyi Biotec
**Western blot**	FOXP3	PCH101	14-4776-82	none	eBioscience, ThermoFisher Scientific, Les Ulis, France
	FOXP3	D608R	12632S	none	Cell Signaling Technologies, Ozyme, Saint-Cyr-l'Ecole, France
	STAT3	D3Z2G	12640S	none	Cell Signaling Technologies
	$pSer^{727}$ STAT3	D8C2Z	94994S	none	Cell Signaling Technologies
	$pTyr^{705}$ STAT3	D3A7	9145S	none	Cell Signaling Technologies
	Smad2/Smad3	D7G7	8685S	none	Cell Signaling Technologies
	$pSer^{465/467}$ Smad2/ $pSer^{423/425}$ Smad3	D27F4	8828S	none	Cell Signaling Technologies
	GAPDH	411	sc-47724	none	Santa Cruz Biotechnology, Heidelberg, Germany
	β-actin	AC-74	A5316	none	Sigma Aldrich, Merck, Darmstadt, Germany
	β-tubulin	AA2	05-661-I	none	Sigma Aldrich
**Flow Cytometry**	CD3*&	UCHT1	555332	FITC	BD Biosciences, Pont-de-Claix, France
	CD4*&	RPA-T4	560650	PerCPCy5.5	BD Biosciences
Immune cells in isolated PBMCs	CD5*	UCHT2	563516	APC-Cy7	BD Biosciences
* 1st panel	CD8&	SK1	557834	APC-Cy7	BD Biosciences
& 2nd panel	CD14*	MΦP9	563743	BV421	BD Biosciences
	CD16*	B73.1	561304	APC	BD Biosciences

	CD19*[&]	SJ25C1	557835	PE-Cy7	BD Biosciences
	CD56*	MY31	556647	PE	BD Biosciences
**Flow Cytometry**	CD19	HIB19	561121	V500	BD Biosciences
	CD5	UCHT2	555352	FITC	BD Biosciences
B/CD4$^+$ T	CD25	M-A251	557753	APC-Cy7	BD Biosciences
FOXP3	CD127	HIL-7R-M21	560822	PE-Cy7	BD Biosciences
	FOXP3	259D/C7	560046	PE	BD Biosciences
**Flow Cytometry**	CD3	UCHT1	555332	FITC	BD Biosciences
	CD5	UCHT2	563516	APC-Cy7	BD Biosciences
PBMCs	CD19	SJ25C1	557835	PE-Cy7	BD Biosciences
IL10/TGFβ1	IL10	JES3-19F1	554707	APC	BD Biosciences
	TGFβ1	TW4-9E7	562423	PerCP-Cy5.5	BD Biosciences
**Flow Cytometry**	CD19	HIB19	561121	V500	BD Biosciences
	CD5	UCHT2	561154	V450	BD Biosciences
	CD24	ML5	555427	FITC	BD Biosciences
B cells	CD25	M-A251	557753	APC-Cy7	BD Biosciences
IL10/TGFβ1	CD27	M-T271	560612	PerCPCy5.5	BD Biosciences
	CD38	HIT2	560677	PE-Cy7	BD Biosciences
	IL10	JES3-19F1	554707	APC	BD Biosciences
	TGFβ1	TW4-9E7	562339	PE	BD Biosciences
**Flow Cytometry**	CD19	LT19	130-113-170	PEVio770	Miltenyi Biotec
	CD3	REA613	130-113-141	PerCPVio700	Miltenyi Biotec
	CD5	UCHT2	130-119-852	APCVio770	Miltenyi Biotec
B/CD4$^+$ T IFNγ/TNFα	IFNγ	B27	559327	PE	BD Biosciences
	Mouse IgG1, κ	MOPC-21	551436	PE	BD Biosciences
	TNFα	cA2	130-120-490	FITC	Miltenyi Biotec
	REA Control IgG1	REA293	130-113-449	FITC	Miltenyi Biotec
**Flow Cytometry**	CD3	SK7	557832	APC-H7	BD Biosciences
	CD4	SK3	566104	BV480	BD Biosciences
	CD5	L17F12	751278	BUV615	BD Biosciences
PBMCs	CD8	RPA-T8	563795	BUV395	BD Biosciences
Anti-IL10 or TGFβRI	CD19	SJ25C1	563325	BV786	BD Biosciences
	CD25	M-A251	561398	Alexa Fluor 700	BD Biosciences
	CD127	HIL-7R-M21	566398	BB700	BD Biosciences

	IFNγ	4S.B3	560741	PE-Cy7	BD Biosciences
	Mouse IgG1 κ	MOPC-21	557872	PE-Cy7	BD Biosciences
	IL4	8D4-8	560671	APC	BD Biosciences
	Mouse IgG1 κ	MOPC-21	554681	APC	BD Biosciences
**Flow Cytometry**	CD3	SK7	741206	BUV496	BD Biosciences
	CD5	L17F12	751278	BUV615	BD Biosciences
	CD25	M-A251	557741	PE-Cy7	BD Biosciences
PBMCs	CD19	SJ25C1	557791	APC-Cy7	BD Biosciences
IL10/TGFβ1/FOXP3	CD4	SK3	566104	BV480	BD Biosciences
	CD27	L128	612829	BUV737	BD Biosciences
Cohort #2	CD127	HIL-7R-M21	563324	BV786	BD Biosciences
	IL10	JES3-19F1	554707	APC	BD Biosciences
	Rat IgG2a κ	R35-95	551139	APC	BD Biosciences
	TGFβ1	TW4-9E7	562423	PerCP-Cy5.5	BD Biosciences
	Mouse IgG1 κ	MOPC-21	550795	PerCP-Cy5.5	BD Biosciences
	FOXP3	PCH101	53-4776-42	Alexa Fluor 488	eBiosciences
	Rat IgG2a κ	eBR2a	53-4321-80	Alexa Fluor 488	eBiosciences
**Flow Cytometry**	CD19	SJ25C1	557835	PE-Cy7	BD Biosciences
	CD5	UCHT2	562646	BV421	BD Biosciences
	CD3	UCHT1	561416	V500	BD Biosciences
B cells	IL10 RNA probe		VA6-13016-PF	Alexa Fluor 750	Invitrogen,Thermo Fisher Scientific, Les Ulis, France
IL10/TGFβ1 mRNA	TGFβ1 RNA probe		VA4-18704-PF	Alexa Fluor 488	Invitrogen
**Flow Cytometry**	CD3	SK7	557832	APC-Cy7	BD Biosciences
	CD4	SK3	566910	PE	BD Biosciences
	CD5	L17F12	751278	BUV615	BD Biosciences
PBMCs	CD19	SJ25C1	566396	BB700	BD Biosciences
mRNA/protein FOXP3	CD25	M-A251	557741	PE-Cy7	BD Biosciences
	CD27	M-T271	741833	BUV737	BD Biosciences
	CD127	HIL-7R-M21	563324	BV786	BD Biosciences
	FOXP3	PCH101	53-4776-42	Alexa Fluor 488	eBiosciences
	Rat IgG2a κ	eBR2a	53-4321-80	Alexa Fluor 488	eBiosciences
	FOXP3 RNA probe		VA1-15518-PF	Alexa Fluor 647	Invitrogen

Supplementary Table 4: MFI of the CD markers used for Figures 2D and S2D

Unstimulated

UPN	CD5 IL10-	CD5 IL10+	CD19 IL10-	CD19 IL10+	CD27 IL10-	CD27 IL10+	CD24 IL10-	CD24 IL10+	CD25 IL10-	CD25 IL10+	CD38 IL10-	CD38 IL10+
**142**	509	1420	1020	1610	456	2640	938	1338	189	310	705	840
**365**	884	1950	1310	2340	554	1780	652	570	390	564	447	451
**323**	460	605	989	1180	1200	2100	1188	1408	144	90	2250	3515
**377**	726	803	770	851	418	478	652	662	234	238	118	68
**341**	309	546	966	1130	308	398	1700	1090	230	244	413	351
**376**	312	1060	1530	2810	842	6020	1798	1334	312	454	1740	4940
**237**	266	533	1870	1820	226	828	568	456	1052	324	2215	76
**164**	779	1990	905	1230	438	1776	1042	1566	266	274	735	985
**58**	518	1490	727	1020	240	654	818	1088	139	104	247	116
**187**	1780	3130	1830	1980	1164	2340	1782	2280	380	135	570	209
**126**	ND	ND	ND	ND	ND	ND	ND	ND	ND	ND	ND	ND
**201**	3690	6380	2170	3440	2040	3240	1112	942	390	554	416	1255
**232**	1170	1260	1300	1560	588	778	1420	1670	254	426	620	1135
**390**	706	560	1110	1150	518	530	1400	1226	119	122	555	725
**307**	1980	2410	2690	2530	928	1232	1702	1206	684	660	1290	1340
**167**	1410	1700	1050	1380	1742	2440	792	1090	400	ND	ND	ND
**124**	1900	1620	6440	2620	574	564	910	558	2260	510	6250	323

Stimulated

UPN	CD5 IL10-	CD5 IL10+	CD19 IL10-	CD19 IL10+	CD27 IL10-	CD27 IL10+	CD24 IL10-	CD24 IL10+	CD25 IL10-	CD25 IL10+	CD38 IL10-	CD38 IL10+
**142**	425	597	989	1230	600	1894	764	704	139	224	590	540
**365**	698	879	1000	1270	498	968	640	684	246	274	347	178
**323**	384	397	918	1000	434	476	806	766	176	119	1715	2145
**377**	553	678	737	755	338	326	644	576	210	195	178	72
**341**	260	392	786	1200	224	406	1052	624	191	197	409	326
**376**	254	482	1250	1900	828	1270	1032	712	254	364	1315	2445
**237**	277	275	1090	1070	191	220	336	392	324	350	484	320
**164**	296	363	1050	1580	191	320	330	344	200	264	136	61
**58**	530	1360	528	700	332	1570	514	684	195	177	365	590
**187**	2420	3290	1820	2200	1340	1796	2200	2400	202	146	231	173
**126**	ND	ND	ND	ND	ND	ND	ND	ND	ND	ND	ND	ND
**201**	2430	4320	1920	3140	1428	2340	934	888	314	464	380	1120
**232**	1010	1100	901	991	446	602	986	1124	165	184	328	453
**390**	621	560	850	1010	450	612	1016	1056	83	155	453	540
**307**	2030	2830	3020	3650	720	962	1122	1164	736	1092	935	1465
**167**	479	501	1350	952	386	412	642	788	1870	1012	91	87
**124**	1690	1350	4600	2130	578	494	886	618	2240	630	6700	238

Unstimulated

UPN	CD5 TGFβ1-	CD5 TGFb1+	CD19 TGFβ1-	CD19 TGFb1+	CD27 TGFβ1-	CD27 TGFb1+	CD24 TGFβ1-	CD24 TGFb1+	CD25 TGFβ1-	CD25 TGFb1+	CD38 TGFβ1-	CD38 TGFb1+
**142**	836	1660	1260	1770	1442	3040	1168	1312	242	344	675	985
**365**	1150	1480	1460	2610	730	1560	612	612	440	552	451	445
**323**	473	523	985	1180	1040	3320	1108	1754	133	139	2280	3275
**377**	676	881	759	857	386	566	624	728	197	322	121	66
**341**	315	363	911	1380	284	648	1602	2300	234	220	450	245
**376**	542	541	1580	2270	424	4200	1256	1752	478	366	3145	2925
**237**	359	365	1830	1910	458	492	502	698	372	552	216	675
**164**	1780	2330	1140	1660	1528	2260	1518	1304	260	364	860	1540
**58**	587	710	792	898	282	378	864	960	131	170	215	292
**187**	1610	3100	1840	1960	914	2420	1612	2380	432	153	645	219
**126**	765	1100	1960	2960	690	950	1556	2480	432	342	494	108
**201**	2530	4940	1560	2630	1106	2780	1152	1052	312	460	368	525
**232**	1160	1410	1290	1910	586	1254	1416	2040	254	276	620	710
**390**	705	845	1110	1590	516	932	1400	1744	120	94	555	660
**307**	2030	2160	2650	3570	956	1352	1626	1184	680	638	1295	1430
**167**	1350	1790	1030	1230	1696	2060	786	834	390	462	51	51
**124**	1860	2740	6430	2620	562	1002	908	468	2260	740	6250	312

Stimulated

UPN	CD5 TGFβ1-	CD5 TGFb1+	CD19 TGFβ1-	CD19 TGFb1+	CD27 TGFβ1-	CD27 TGFb1+	CD24 TGFβ1-	CD24 TGFb1+	CD25 TGFβ1-	CD25 TGFb1+	CD38 TGFβ1-	CD38 TGFb1+
**142**	500	604	1070	1260	908	2080	742	700	172	228	478	580
**365**	677	811	971	1150	400	822	714	622	254	254	750	146
**323**	457	288	1120	690	462	378	940	594	228	106	2205	955
**377**	639	461	794	665	330	348	646	600	222	187	204	85
**341**	262	274	764	1130	220	344	1062	770	195	164	436	191
**376**	420	364	1180	1570	468	1146	1158	790	312	304	2435	1825
**237**	328	371	1340	1790	248	372	338	380	234	424	91	113
**164**	1010	1430	597	752	830	2060	706	606	178	185	481	615
**58**	ND	ND	ND	ND	ND	ND	ND	ND	ND	ND	ND	ND
**187**	1810	3080	1820	2030	946	1752	1838	2460	300	151	383	165
**126**	ND	ND	ND	ND	ND	ND	ND	ND	ND	ND	ND	ND
**201**	2040	3120	1510	2410	1012	1948	968	898	268	378	329	540
**232**	1010	1010	899	1070	444	658	984	1232	166	168	327	387
**390**	620	757	849	1430	450	978	1014	1358	83	89	452	585
**307**	2150	2370	3130	3800	768	972	1134	872	784	1124	1010	1400
**167**	434	740	1080	2470	344	512	612	746	1390	4160	136	51
**124**	1720	1230	4690	1970	582	480	896	514	2340	582	7100	196
